# Supplementary material for: Associations between autistic traits and early ear and upper respiratory signs: a prospective observational study of the Avon Longitudinal Study of Parents and Children (ALSPAC) geographically defined childhood population
Source: BMJ Open. 2023 Feb 21;13(3):e067682. doi: 10.1136/bmjopen-2022-067682 (PMC10186415; doi:10.1136/bmjopen-2022-067682)
Supplement: Supplementary data [file bmjopen-2022-067682supp001.pdf]

Hearing and autism: Supplementary Tables

Supplementary Table 1. Comparison of the prevalence of **mouth breathing** among index children (i.e. with high levels of autistic traits or diagnosed autism) and control children (i.e. all who were not in the index category)

Age		SCDC % (n)	Coherence % (n)	Sociability % (n)	Repetitive % (n)	Autism % (n)
18m	Index	13.1 (117)	15.1 (99)	15.1 (143)	13.7 (289)	17.4 (24)
	Control	10.2 (627)	10.5 (633)	11.4 (875)	9.9 (540)	12.2 (1196)
	P	**	****	****	****	*
30m	Index	27.2 (232)	26.5 (169)	24.5 (226)	24.1 (489)	30.3 (42)
	Control	19.1 (1119)	19.8 (1145)	20.8 (1524)	19.0 (992)	21.8 (1966)
	P	****	****	*	****	**
42m	Index	26.9 (260)	25.4 (185)	23.3 (250)	24.9 (573)	25.5 (38)
	Control	18.6 (1219)	18.9 (1228)	20.3 (1690)	18.8 (1013)	20.7 (2054)
	P	****	****	*	****	-

*P< 0.05; ** P < 0.01; *** P < 0.001; **** P < 0.0001

Supplementary Table 2. Comparison of the prevalence of **snoring** among index children (i.e. with high levels of autistic traits or diagnosed autism) and control children (i.e. all who were not in the index category)

Age		SCDC % (n)	Coherence % (n)	Sociability % (n)	Repetitive % (n)	Autism % (n)
18m	Index	15.3 (144)	14.6 (102)	11.8 (122)	11.5 (258)	16.3 (24)
	Control	7.6 (495)	7.9 (511)	8.8 (719)	7.3 (427)	9.5 (998)
	P	****	****	**	****	*
30m	Index	25.4 (221)	22.0 (150)	20.9 (196)	20.2 (417)	31.0 (41)
	Control	16.0 (947)	16.6 (967)	17.4 (1283)	16.1(851)	18.2 (1660)
	P	****	****	**	****	***
42m	Index	24.6 (238)	23.2 (169)	22.2 (237)	22.0 (506)	26.2 (39)
	Control	17.6 (1152)	17.9 (1158)	18.5 (1534)	17.3 (1020)	19.1 (1889)
	P	****	****	**	****	*

*P< 0.05; ** P < 0.01; *** P < 0.001; **** P < 0.0001

Supplementary Table 3. Comparison of the prevalence of possible **sleep apnoea** among index children (i.e. with high levels of autistic traits or diagnosed autism) and control children (i.e. all who were not in the index category).

Age		SCDC % (n)	Coherence % (n)	Sociability % (n)	Repetitive % (n)	Autism % (n)
18m	Index	10.8 (91)	8.8 (54)	8.2 (77)	9.3 (189)	9.9 (13)
	Control	6.8 (405)	7.0 (409)	7.9 (590)	6.9 (367)	7.9 (769)
	P	****	*	-	**	-
30m	Index	18.1 (148)	18.4 (110)	15.6 (143)	17.4 (339)	16.8 (21)
	Control	14.0 (802)	13.9 (789)	15.2 (1099)	13.6 (705)	15.5 (1387)
	P	***	***	-	****	-
42m	Index	16.4 (158)	17.4 (126)	13.8 (148)	15.4 (355)	18.8 (28)
	Control	12.2 (799)	12.1 (784)	12.9 (1073)	12.0 (709)	14.2 (1298)
	P	***	****	-	****	*

*P< 0.05; ** P < 0.01; *** P < 0.001; **** P < 0.0001

Supplementary Table 4. Comparison of the prevalence of **often pulling and poking at ears** among index children (i.e. with high levels of autistic traits or diagnosed autism) and control children (i.e. all who were not in the index category).

Age		SCDC % (n)	Coherence % (n)	Sociability % (n)	Repetitive % (n)	Autism % (n)
18m	Index	10.4 (103)	10.6 (77)	9.3 (99)	9.8 (226)	10.9 (17)
	Control	6.9 (460)	6.9 (456)	7.5 (634)	6.6 (395)	7.9 (862)
	P	****	****	*	****	-
30m	Index	8.2 (78)	8.4 (60)	6.8 (71)	7.0 (157)	16.2 (25)
	Control	4.1 (267)	4.2 (268)	4.4 (353)	3.7 (213)	4.6 (463)
	P	****	****	**	****	****
42m	Index	1.8 (17)	2.8 (20)	2.2 (24)	2.5 (58)	10.7 (16)
	Control	1.8 (116)	1.8 (115)	2.0 (163)	1.8 (106)	5.3 (521)
	P	-	-	-	*	***

*P< 0.05; ** P < 0.01; *** P < 0.001; **** P < 0.0001

Supplementary Table 5. Comparison of the prevalence of **ears going red and sore looking for a long time** among index children (i.e. with high levels of autistic traits or diagnosed autism) and control children (i.e. all who were not in the index category).

Age		SCDC	Coherence	Sociability	Repetitive	Autism
18m	Index	27.3 (261)	27.4 (194)	24.9 (259)	26.6 (602)	25.3 (38)
	Control	19.6 (1290)	19.9 (1292)	21.0 (1736)	18.3 (1072)	21.6 (2298)
	P	****	****	**	****	-
30m	Index	21.0 (221)	20.4 (139)	18.0 (181)	19.2 (418)	26.5 (40)
	Control	13.0 (820)	13.5 (841)	14.6 (1150)	12.1(680)	15.0 (1458)
	P	****	****	**	****	***
42m	Index	17.0 (164)	17.7 (129)	14.7 (158)	16.1 (371)	19.5 (29)
	Control	12.1 (792)	12.0 (779)	12.9 (14)	11.3 (670)	13.2 (1303)
	P	****	****	**	****	*

*P< 0.05; ** P < 0.01; *** P < 0.001; **** P < 0.0001

Supplementary Table 6. Comparison of the prevalence of **pus/sticky mucus leaking from ears more than once** among index children (i.e. with high levels of autistic traits or diagnosed autism) and control children (i.e. all who were not in the index category).

Age		SCDC<br>% (n)	Coherence<br>% (n)	Sociability<br>% (n)	Repetitive<br>% (n)	Autism<br>% (n)
18m	Index	2.8 (27)	4.7 (34)	3.8 (40)	3.2 (73)	5.3 (8)
	Control	2.8 (184)	2.6 (169)	3.0 (250)	2.7 (159)	3.0 (322)
	P	-	***	-	-	-
30m	Index	3.9 (37)	5.1 (36)	4.4 (46)	4.0 (90)	10.3 (16)
	Control	3.2 (207)	3.0 (189)	3.3 (263)	3.1(177)	6.6 (349)
	P	-	**	-	-	***
42m	Index	8.3 (80)	10.3 (75)	7.2 (77)	7.3 (167)	15.4 (23)
	Control	5.2 (342)	5.2 (339)	5.7 (471)	5.3 (311)	5.8 (575)
	P	****	****	-	****	****
3-4 y	Index	4.8 (44)	4.7 (33)	5.6 (55)	4.0 (90)	9.6 (14)
	Control	3.2 (207)	3.2 (200)	3.2 (245)	3.1 (181)	3.5 (324)
	P	**	**	**	-	**

*P< 0.05; ** P < 0.01; *** P < 0.001; **** P < 0.0001

Supplementary Table 7. Comparison of the prevalence of **hearing reported to be worse during a cold** among index children (i.e. with high levels of autistic traits or diagnosed autism) and control children (i.e. all who were not in the index category).

**Age**		**SCDC % (n)**	**Coherence % (n)**	**Sociability % (n)**	**Repetitive % (n)**	**Autism % (n)**
18m	Index	20.2 (173)	21.9 (137)	17.7 (165)	18.5 (369)	29.9 (41)
	Control	14.4 (842)	14.6 (844)	15.4 (1143)	13.9 (736)	15.4 (1485)
	P	****	****	-	****	****
30m	Index	21.5 (148)	25.8 (133)	16.5 (127)	20.1 (319)	37.9 (44)
	Control	14.1 (682)	14.1 (673)	15.6 (946)	13.1(574)	15.2 (1136)
	P	****	****	-	****	****
42m	Index	37.1 (354)	41.4 (297)	31.6 (335)	36.3 (827)	52.4 (77)
	Control	29.9 (1943)	29.3 (1880)	30.5 (2512)	28.6 (1676)	30.5 (2989)
	P	****	****	-	****	****

*P< 0.05; ** P < 0.01; *** P < 0.001; **** P < 0.0001

Supplementary Table 8. Comparison of the prevalence of **rarely listening** among index children (i.e. with high levels of autistic traits or diagnosed autism) and control children (i.e. all who were not in the index category).

**Age**		**SCDC % (n)**	**Coherence % (n)**	**Sociability % (n)**	**Repetitive % (n)**	**Autism % (n)**
18m	Index	4.2 (42)	3.9 (29)	3.8 (40)	3.0 (70)	5.1 (8)
	Control	1.6 (108)	1.6 (103)	1.9 (162)	1.7 (98)	2.1 (235)
	P	****	****	*	*	*
30m	Index	4.6 (44)	6.4 (45)	5.1 (53)	4.1 (91)	14.4 (22)
	Control	1.6 (101)	1.5 (95)	1.7 (139)	1.4 (80)	2.1 (213)
	P	****	****	****	****	****
42m	Index	7.7 (75)	8.9 (65)	6.7 (72)	5.5 (126)	20.2 (30)
	Control	2.5 (165)	2.3 (148)	2.8 (235)	2.3 (135)	3.1 (306)
	P	****	****	****	****	****

*P< 0.05; ** P < 0.01; *** P < 0.001; **** P < 0.0001
