# Supplementary material for: Associations between autistic traits and early ear and upper respiratory signs: a prospective observational study of the Avon Longitudinal Study of Parents and Children (ALSPAC) geographically defined childhood population
Source: BMJ Open. 2023 Feb 21;13(3):e067682. doi: 10.1136/bmjopen-2022-067682 (PMC10186415; doi:10.1136/bmjopen-2022-067682)
Supplement: Author's manuscript [file bmjopen-2022-067682.draft_revisions.pdf]

BMJ Open is committed to open peer review. As part of this commitment we make the peer review history of every article we publish publicly available.

When an article is published we post the peer reviewers' comments and the authors' responses online. We also post the versions of the paper that were used during peer review. These are the versions that the peer review comments apply to.

The versions of the paper that follow are the versions that were submitted during the peer review process. They are not the versions of record or the final published versions. They should not be cited or distributed as the published version of this manuscript.

BMJ Open is an open access journal and the full, final, typeset and author-corrected version of record of the manuscript is available on our site with no access controls, subscription charges or pay-per-view fees (http://bmjopen.bmj.com).

If you have any questions on BMJ Open's open peer review process please email info.bmjopen@bmj.com

BMJ Open

Associations between autistic traits and early ear and upper respiratory signs: a prospective observational study of the ALSPAC geographically defined childhood population

Journal:	*BMJ Open*
Manuscript ID	bmjopen-2022-067682
Article Type:	Original research
Date Submitted by the Author:	25-Aug-2022

Complete List of Authors:	Hall, Amanda; Aston University, Life and Health Sciences<br>Maw, Richard; University of Bristol Faculty of Medicine and Dentistry, St Michael’s Hospital<br>Iles-Caven, Yasmin; University of Bristol Faculty of Medicine and Dentistry, Bristol Medical School (PHS)<br>Gregory, Steven; University of Bristol Faculty of Medicine and Dentistry, Bristol Medical School (PHS)<br>Rai, Dheeraj; University of Bristol Faculty of Medicine and Dentistry, Centre for Academic Mental Health<br>Golding, Jean; University of Bristol Faculty of Medicine and Dentistry, Bristol Medical School (PHS)

Keywords:	Paediatric otolaryngology < OTOLARYNGOLOGY, EPIDEMIOLOGY, Audiology < OTOLARYNGOLOGY, Delirium & cognitive disorders < PSYCHIATRY

SCHOLARONE™
Manuscripts

I, the Submitting Author has the right to grant and does grant on behalf of all authors of the Work (as defined in the below author licence), an exclusive licence and/or a non-exclusive licence for contributions from authors who are: i) UK Crown employees; ii) where BMJ has agreed a CC-BY licence shall apply, and/or iii) in accordance with the terms applicable for US Federal Government officers or employees acting as part of their official duties; on a worldwide, perpetual, irrevocable, royalty-free basis to BMJ Publishing Group Ltd ("BMJ") its licensees and where the relevant Journal is co-owned by BMJ to the co-owners of the Journal, to publish the Work in this journal and any other BMJ products and to exploit all rights, as set out in our licence.

The Submitting Author accepts and understands that any supply made under these terms is made by BMJ to the Submitting Author unless you are acting as an employee on behalf of your employer or a postgraduate student of an affiliated institution which is paying any applicable article publishing charge ("APC") for Open Access articles. Where the Submitting Author wishes to make the Work available on an Open Access basis (and intends to pay the relevant APC), the terms of reuse of such Open Access shall be governed by a Creative Commons licence – details of these licences and which Creative Commons licence will apply to this Work are set out in our licence referred to above.

Other than as permitted in any relevant BMJ Author's Self Archiving Policies, I confirm this Work has not been accepted for publication elsewhere, is not being considered for publication elsewhere and does not duplicate material already published. I confirm all authors consent to publication of this Work and authorise the granting of this licence.

For peer review only

BMJopen 22.8.22 Final

Associations between autistic traits and early ear and upper respiratory signs: a prospective observational study of the ALSPAC geographically defined childhood population

Amanda Hall[1] (0000-0001-8520-6005), **Richard Maw**[2], **Yasmin Iles-Caven**[3](0000-0002-9965-9133), **Steven Gregory**[3](0000-0002-1529-9338), **Dheeraj Rai**[4] (0000-0002-7239-3523) **and Jean Golding**[3] (0000-0003-2826-3307)

[1] College of Health and Life Sciences, Aston University, Birmingham, UK, Amanda Hall Senior Lecturer in Audiology; [2]University Hospitals Bristol NHS Foundation Trust, Richard Maw retired NHS Consultant ENT Surgeon; [3]Centre for Academic Child Health, Population Health Sciences, Bristol Medical School, University of Bristol, Bristol BS8 2BN, UK Yasmin Iles-Caven, Senior Research Associate, Steven Gregory Senior Data Manager, Jean Golding, Emeritus Professor of Paediatric and Perinatal Epidemiology; [4]Centre for Academic Mental Health, Bristol Medical School, University of Bristol, Dheeraj Rai Associate Professor in Neurodevelopmental Psychiatry.

Correspondence to J Golding (jean.golding@bristol.ac.uk):

Centre for Academic Child Health,
Bristol Medical School (Population Health Sciences)
University of Bristol
Oakfield House
Oakfield Grove
Bristol BS8 2BN, U.K.

For peer review only

Abstract

Objective: To determine whether early ear and upper respiratory signs are associated with the development of high levels of autistic traits or diagnosed autism.

Design: Longitudinal birth cohort: Avon Longitudinal Study of Parents and Children (ALSPAC).

Setting: Area centred on the city of Bristol in Southwest England. Eligible pregnant women resident in the area with expected date of delivery between April 1991 and December 1992 inclusive.

Participants: 10,000+ young children followed throughout their first 4 years. Their mothers completed three questionnaires between 18-42 months recording the frequency of nine different signs and symptoms relating to the upper respiratory system, as well as ear and hearing problems.

Outcome measures: Primary – high levels of autism traits (social communication, coherent speech, sociability, and repetitive behaviour); secondary – diagnosed autism.

Results: Early evidence of mouth breathing, snoring, pulling/ poking ears, ears going red, hearing worse during a cold, and rarely listening were associated with high scores on each autism trait and with a diagnosis of autism. There was also evidence of associations of pus or sticky mucus discharge from ears especially with autism and with poor coherent speech. Adjustment for ten environmental characteristics made little difference to the results, and substantially more adjusted associations were at P<0.001 than expected by chance (41 observed; 0.01 expected). For example, for discharge of pus or sticky mucus from ears the adjusted odds ratio (AOR) for autism at 30 months was 3.29 [95%CI:1.85,5.86; P<0.001], and for impaired hearing during a cold AOR=2.18 [1.43,3.31; P<0.001].

Conclusions: Very young children exhibiting common ear and upper respiratory signs appear to have an increased risk of a subsequent diagnosis of autism or demonstrated high levels of autism traits. Results suggest the need for identification and management of ENT conditions in autistic children and may provide possible indicators of causal mechanisms.

Keywords: ALSPAC; autism; early childhood; upper respiratory symptoms; hearing; snoring

For peer review only

Strengths and difficulties of this study

- The strength of these results lies in the fact that the data were collected prospectively using a geographically defined population of young children.
- Two of the autism traits were identified using well validated scales (social communication and speech coherence).
- There was no bias in the way in which the questions were asked nor in the later identification of autism or of autism traits.
- The disadvantage was that not all children in the study were assessed for a diagnosis of autism.

Introduction

Autism spectrum disorder, henceforward named as 'autism' is a fairly uncommon, yet heterogeneous, condition.[1] Its aetiology is complex and likely to involve a combination of gene-environment-biology interactions, with no one single cause.[2] There is increasing recognition that the aetiology of each autistic trait may differ, either genetically, epigenetically and/or conditionally upon an environmental exposure.[2]

Understanding and addressing early comorbidities of children diagnosed with autism may lead to longer term improvements in quality of life and also inform the common mechanisms and pathways in the origins of autism.[3] Ear, nose and throat (ENT) including related hearing disorders have been implicated in the development of autism[4 5] including acute otitis media (ear infections), otitis media with effusion (OME or glue ear), temporary conductive hearing loss associated with otitis media, and sleep disordered breathing. For example, there have been a number of studies using population health records, comparing the frequency of ear infections in children with autism compared to controls. Most showed greater odds of ear infections in children with autism: for example, a US population sample of 483 autism children with 84,789 controls found an odds ratio (OR) of 3.1 (95% CI 2.1 to 4.7) for at least three ear infections;[6] others have also shown excesses of ear infections.[7 8] Conversely two studies using Kaiser Permanente health records[9] showed no excess of ear infections among children with autism, but one did find an increased odds (OR 1.93, 95 CI 1.56 to 2.38) of ENT conditions associated with autism compared to controls.[5] Other conditions that have been implicated include sleep-disordered breathing or obstructive sleep apnoea in the early life history of children with autism in the USA[5 10] and Japan.[11] Others have shown an increased likelihood of surgery for a sleep disorder, such as adenoidotonsillectomy.[12]

Of other ENT conditions, there are fewer studies examining early history of chronic OME which is more likely than acute otitis media to interfere with hearing, although less easy to identify from health records. One method is to examine the rate of insertion of ventilation tubes, although it is not always possible to distinguish ventilation tubes inserted for OME from insertion for acute ear infections. Nevertheless, ventilation tube rates have been shown to be higher in children with autism.[7 13 14] Case control studies comparing auditory function in children with autism have found more abnormal tympanograms in children suggesting a higher prevalence of OME,[15] and more auditory abnormalities.[16] Although other studies did not find more auditory abnormalities in children with autism[17 18];

For peer review only

in both these studies children were excluded if they had a history of ear infections and middle ear effusion.

Overall, therefore, the evidence suggests an increased prevalence of ENT and related hearing conditions in children with autism compared with typically developing children. Much of the evidence cited above is from health records which may be biased due to greater levels of help-seeking from health services of parents of children with suspected autism. Where a parent has concerns about their child's general development, they may be more likely to attend health services, resulting in increased recording of the presence of common health conditions even where the underlying prevalence is no more common than in the general population.

Longitudinal population cohort studies avoid many of the biases in studies using health records, but there are few such studies in the literature apart from that of Jeans and colleagues[19] who analysed data from the Early Childhood Longitudinal Study Birth cohort and found an increased OR of 1.46 [95% CI 1.24, 1.72] of early ear infections in children with a later autism diagnosis. The present study, positioned within the longitudinal birth cohort ALSPAC (Avon Longitudinal Study of Parents and Children), uses information collected prospectively from birth to assess whether any early indications of ENT symptoms, including ear infections and intermittent hearing problems, are associated with one or more of the traits shown to be strongly related to a diagnosis of autism. Its aim is to investigate whether children with autism, and those with high levels of autistic traits or a diagnosis of autism were recorded as having more early ear and upper respiratory signs and symptoms than expected by chance.

Material and methods

The study population

ALSPAC is a pre-birth cohort study that enrolled ~80% of pregnant women resident in the Avon area of the UK in 1991-2. The initial number of pregnancies enrolled was 14,541 (for these at least one questionnaire had been returned or a "Children in Focus" clinic had been attended by 19/07/99). Of these initial pregnancies, there was a total of 14,676 fetuses, resulting in 14,062 live births and 13,988 children who were alive at 1 year of age. The aim of the study is to assess ways in which the environment (defined in its broadest sense) interacts with genetics to influence the health, development and well-being of the offspring.[20] To this end, data collection used a variety of methodologies including direct examination of the offspring, self-completion questionnaires administered to the parents, the children and their teachers, collection and assays of biological samples (including DNA), linkage to health and education records.[21 22] The study website contains details of all the data that is available through a fully searchable data dictionary and variable search tool: http://www.bristol.ac.uk/alspac/researchers/our-data/. ALSPAC has had its own ethics committee since its inception.[23]

Patient and public involvement

Patients and/or the public were not involved in the reporting or dissemination plans of this research. Prior to data collection (in the early 1990s), focus groups of pregnant women were involved in discussions on the content of questionnaires.

For peer review only - http://bmjopen.bmj.com/site/about/guidelines.xhtml

The Outcomes

Identification of autism

In order to identify the children with autism we used the following sources: (a) a review of all children given a statement for special educational provision in the Avon area, to identify children diagnosed by age 11 as having special educational needs conforming to a diagnosis of autism using the ICD-10 criteria[24]; (b) the mother's answer to the question at age 9 "Have you ever been told that your child has autism, Asperger's syndrome or autistic spectrum disorder"; (c) classification as Pervasive Development Disorder using questions from the DAWBA questionnaire at 91 months,[25] with the answers to the questionnaire classified by a child psychiatrist; (d) text responses to any question on diagnoses given to the child in questionnaires from 6 months to 11 years suggesting the child had been given an autism diagnosis; (e) *ad hoc* letters from parents to the Study Director. We considered that no one of these sources would be adequate, and so used all, and monitored the overlaps. In this way we identified 177 ALSPAC offspring with a probable diagnosis of autism – 139 boys and 38 girls. Validation of the use of this group as autism was shown by its strong relationship with a polygenic risk score for autism.[26]

Autistic traits

We have used the upper 10th centile of the scores on the four independent trait predictors of autism identified previously as most predictive of autism in this cohort and described below. They included measures of social communication, coherent speech, sociability temperament and repetitive behaviour. Each had been shown to be an independent predictor of autism as identified using clinical records in this cohort.[27]

The Social Communication Trait

We used the 12-item Social and Communication Disorders Checklist (SCDC), developed by Skuse and colleagues.[28] They showed that the internal consistency was excellent (0.93) and the test-retest reliability was high (0.81). The method was developed on clinical samples, and when later used on the ALSPAC population at age 7.7 years the high end of the scale was shown to predict a variety of adverse outcomes, but was most specific for autism spectrum disorder.[29] Further research with ALSPAC data showed that the measure was reasonably stable over time.[30] For the present analysis we have used the prorated score, which was calculated when any items of the scale were missing a response, by using the average of the items that had been answered by the individual (2.7% of the population, almost all of whom had just one item missing). If all items were missing, the score was put to missing. The measure ranged from 0 to 24, the higher the score the more impaired was the child's social cognition. The distribution was skewed with a long upper tail (12.8% had a score of over 6 and comprise the group with highest autistic trait for these analyses).

The Coherence Measure

At age 9 the study mother completed a questionnaire which included 7 of the 9 scales of the first version of the Children's Communication Checklist (CCC).[31] This checklist was designed to assess aspects of communication that were not readily identified by conventional standardised tests, including aspects of speech and syntax as well as pragmatic aspects such as over-literal interpretation of stereotyped language. Although the CCC was initially designed to identify pragmatic difficulties, it has been shown to be good at discriminating a

For peer review only

wide range of language and communication problems from typical development.[32] Analyses of traits predictive of autism in ALSPAC showed that the Coherence scale performed better than the other CCC scales[27] and consequently it is used here. The scale comprises 8 items (e.g., “It is sometimes hard to make sense of what she is saying because it seems illogical or disconnected” and “She has difficulty in telling a story or describing what she has done in a sequence of events”). The score ranged from 20 to 36, with higher scores indicating more typical behaviour. The score had a skewed distribution. The lower tail used in this analysis comprised those children scoring <33 points (10.0% of the population).

Abnormal and Repetitive Behaviour

This scale was developed from the answer to four questions in the questionnaire sent to the mother at 69 months; these were as follows: “How often does he/she: (a) repeatedly rock his head or body for no reason; (b) have a tic or twitch; (c) have other unusual behaviour”; or (d) “Does he/she stumble or get stuck on words, or repeat them many times? (e.g. I I I I want a sweet)”? The responses to each question were coded as: often/always = 3; sometimes = 2; never = 1 and summed. The resultant scale had a range from 4 to 12, with 22% scoring 5 and only 5.9% scoring more than 5. As it was impossible to approximate to a 10% cut-off, we used > 5 as our group with highest autistic trait.

Sociability Temperament

The questionnaire concerning the child which was sent to the study mothers when the child was 38 months of age included the 20 questions of the EAS Temperament scale[33]; this measured four traits – emotionality, activity, shyness, and sociability – each based on the answers to 5 questions. The range of the Sociability sub-score was from 5 to 25 and the frequency distribution was approximately normal, a high score indicating a high level of sociability. The prorated scale was calculated for missing values as in the scales mentioned above. We then selected the lowest 11.4% of the children for our analyses (score <8) as being the nearest to 10% as the group with highest autistic trait.

Maternal reports of ear and upper respiratory signs and symptoms

Questions regarding their child’s ear and upper respiratory signs were sent to the mother by post when the child was aged: 18, 30 and 42 months. Each questionnaire was structured with response boxes to be ticked. For the current study we used nine questions which were repeated at these three time points, and which described common ENT signs relating to hearing and the upper respiratory system. Details of the actual wording of the questions are shown in Table 1. They cover aspects of breathing (mouth breathing, snoring, symptoms of sleep apnoea), ears (pulling or poking at ears, ears going red, pus discharging from ears), and subjective signs of hearing loss and listening difficulties (hearing worse during a cold, child rarely listens). Questions concerning whether or not the child had had earache were also asked, and the results are provided, but since the children were mostly too young to describe what was wrong, we have treated these data as less reliable than the other questions asked.

For peer review only

Table 1: ENT and hearing related questions in the ALSPAC parental questionnaires

Has anyone thought there may be a problem with his hearing?
Has your baby had earache?
Does s/he breathe through her mouth rather than through her nose?
Does s/he snore for more than a few minutes at a time?
When she is asleep, does s/he seem to stop breathing or hold her breath for several seconds at a time?*
Does she pull, scratch or poke at her ears?
Do his/her ears go red and look sore for a long time?
Has pus or a sticky mucus (not ear wax) ever leaked out of his/her ear?
During or after a cold, is his/her hearing worse than usual?
Generally, does your toddler listen to people or to things that happen nearby?

*indicating sleep apnoea

Potential confounders

Adjustments were made for the following ten potential confounders: preterm gestation (<37 weeks; 37+ weeks), sex, parity (defined as the number of previous pregnancies resulting in a live or stillbirth, 0 v 1+), breast feeding (any v none), maternal depression at 8 weeks post-delivery as assessed using the Edinburgh Postnatal Depression Score,[34] maternal educational achievements (5-point scale), maternal smoking at 18 weeks gestation (none v any), maternal locus of control (using a shortened version of the adult version of the Nowicki-Strickland Internal-External locus of control scale as described elsewhere,[35] child's exposure to environmental tobacco smoke (ETS) at 15 months measured as the length of time the child was in a room with others smoking at weekends and weekdays (any v none) and attending a crèche or other type of day care by 30 months (yes v no). Table 2 shows frequencies of these confounders.

Table 2. Frequencies of variables used as confounders.

**Confounder**	**No. with data available**	**Frequency**
Pre-term delivery	12,935	5.3% <37 weeks
Sex of child	14,676	51.8% male
Parity of mother	12,787	48.2% Primiparous
Whether breast fed at 7 days	11,880	66.1%
Maternal depression at 2m	11,213	8.8%
Maternal education level (5 levels)	12,370	From 20.8% lowest to 12.8% highest
Maternal smoking at 18 weeks gestation	13,274	19.6%
Maternal locus of control	12,471	45.8% external
Child's exposure to environmental tobacco smoke at 15m	11,073	42.0% exposed
Attended creche or equivalent by 3y	10,038	36.3% exposed

For peer review only

Statistical analysis

Initial analysis used unadjusted comparisons between the different signs and symptoms and the five different binary outcomes (the most disadvantaged 10% of the four autistic traits and diagnosed autism). Logistic regression using STATA was then used to allow for the 10 potential confounders – with 120 logistic regressions being undertaken. In order to take account of multiple testing we determined how many associations would be expected to result in P<0.01 by chance for both the unadjusted and the adjusted associations. For each set of analyses just 1.2 of the 120 results were expected at P<0.01. Any excess in numbers of adjusted results at P<0.001 were assumed to be either indicating causality or the possibility of unadjusted confounding.

Results

Unadjusted comparisons of ear and upper respiratory signs with autism outcomes

The responses to each question asked at 18, 30 and 42 months are compared between those scoring at the extreme ends of the autism trait scores, as well as for those diagnosed with autism in Supplementary Tables 1-8. Of the 120 comparisons, 85 were at P<0.01 (1.2 expected) and 66 at P<0.0001 (0.001 expected).

Among the different ages tested, the most striking were at age 30 months (Table 3) where it can be seen that those groups with the highest autistic traits reported more ear and upper respiratory signs. It is noteworthy that autism itself was significantly associated with all signs except for symptoms of sleep apnoea. Conversely, the sociability trait was much less strongly associated with these ear and upper respiratory signs than any of the other three traits. Also note that pus leaking from the ears was only associated with abnormal scores on the speech coherence trait and with autism itself.

Table 3. Summary of **unadjusted** results comparing % rates (n) of ENT signs at 30 months of children who later demonstrated high levels of autistic traits and of children diagnosed with autism contrasted with controls

**Signs and symptoms**	**Group**	**Social communication % (n)**	**Speech Coherence % (n)**	**Sociability %(n)**	**Repetitive behaviour % (n)**	**Autism % (n)**
Mouth breathing	Index	27.2 (232)	26.5 (169)	24.5 (226)	24.1 (489)	30.3 (42)
	Control	19.1 (1119)	19.8 (1145)	20.8 (1524)	19.0 (992)	21.8 (1966)
	P value	***	***	*	***	**
Snoring	Index	25.4 (221)	22.0 (150)	20.9 (196)	20.2 (417)	31.0 (41)
	Control	16.0 (947)	16.6 (967)	17.4 (1283)	16.1(851)	18.2 (1660)
	P value	***	***	**	***	***
Sleep apnoea	Index	18.1 (148)	18.4 (110)	15.6 (143)	17.4 (339)	16.8 (21)
	Control	14.0 (802)	13.9 (789)	15.2 (1099)	13.6 (705)	15.5 (1387)
	P value	***	***		***	

For peer review only

Pulling at ears	Index	8.2 (78)	8.4 (60)	6.8 (71)	7.0 (157)	16.2 (25)
	Control	4.1 (267)	4.2 (268)	4.4 (353)	3.7 (213)	4.6 (463)
	P value	***	***	**	***	***
Ears red and sore	Index	21.0 (221)	20.4 (139)	18.0 (181)	19.2 (418)	26.5 (40)
	Control	13.0 (820)	13.5 (841)	14.6 (1150)	12.1(680)	15.0 (1458)
	P value	***	***	**	***	***
Pus/mucus from ears	Index	3.9 (37)	5.1 (36)	4.4 (46)	4.0 (90)	10.3 (16)
	Control	3.2 (207)	3.0 (189)	3.3 (263)	3.1(177)	6.6 (349)
	P value		**			***
Hearing worse during cold	Index	21.5 (148)	25.8 (133)	16.5 (127)	20.1 (319)	37.9 (44)
	Control	14.1 (682)	14.1 (673)	15.6 (946)	13.1(574)	15.2 (1136)
	P value	***	***		***	***
Rarely listens	Index	4.6 (44)	6.4 (45)	5.1 (53)	4.1 (91)	14.4 (22)
	Control	1.6 (101)	1.5 (95)	1.7 (139)	1.4 (80)	2.1 (213)
	P value	***	***	***	***	***

The asterisks denote positive unadjusted associations with P values as follows:

*P< 0.05; ** P < 0.01; *** P < 0.001

Adjusted comparisons of ear and upper respiratory signs with autism outcomes

Results of adjustment for 10 factors (gestation, sex, parity, breast feeding, maternal depression, education, prenatal smoking, maternal locus of control, child exposure to environmental tobacco smoke, and starting crèche by age 30m) are shown in Table 4. It can be seen that of the 120 logistic regression results, 41 were associated at P<0.001 (0.0012 expected). The results are discussed in terms of the three categories of ear and upper respiratory signs below.

Table 4. High levels of autistic traits or of diagnosed autism by upper respiratory signs. Odds ratios [95% confidence intervals] **adjusted** for gestation, sex, parity, breast feeding, maternal depression, maternal education, prenatal smoking, locus of control, child exposure to environmental tobacco smoke and starting crèche (see text for details).

**Outcome**	**Social communication AOR [95% CI]**	**Speech Coherence AOR [95% CI]**	**Sociability AOR [95% CI]**	**Repetitive behaviour AOR [95% CI]**	**Autism AOR [95% CI]**
<u>Mouth breathing</u>					
18m	1.12 [0.90,1.39]	1.51 [1.21,1.81]***	1.34 [1.07,1.67]**	1.36 [1.14,1.62]***	2.13 [1.32,3.44]**
30m	1.41 [1.20,1.67]***	1.31 [1.10,1.55]**	1.11 [0.93,1.33]	1.24 [1.09,1.43]**	1.62 [1.07,2.43]*
42m	1.43 [1.22,1.67]***	1.32 [1.12,1.57]***	1.09 [0.92,1.30]	1.31 [1.15,1.49]***	1.40 [0.93,2.11]

For peer review only

Snoring					
18m	1.72 [1.23,2.41]**	1.92 [1.35,2.72]***	1.12 [0.76,1.64]	1.31 [0.97,1.77]	1.38 [0.55,3.43]
30m	1.54 [1.18,2.03]**	1.32 [0.98,1.78]	1.13 [0.83,1.53]	1.21 [0.95,1.52]	1.54 [0.77,3.11]
42m	1.47 [1.15,1.88]**	1.33 [1.02,1.73]*	1.00 [0.75,1.33]	1.20 [0.97,1.48]	1.93 [1.09,3.41]*
Sleep apnoea					
18m	1.54 [1.20,1.96]***	1.23 [0.93,1.62]	0.89 [0.66,1.19]	1.33 [1.08,1.63]**	1.14 [0.57,2.28]
30m	1.24 [1.02,1.50]*	1.30 [1.06,1.59]*	0.91 [0.73,1.13]	1.17 [1.00,1.37]*	0.93 [0.53,1.63]
42m	1.28 [1.06,1.55]**	1.33 [1.09,1.62]**	0.99 [0.80,1.23]	1.20 [1.03,1.41]*	1.35 [0.84,2.17]
Pulls/pokes at ears					
18m	1.42 [1.13,1.79]**	1.11 [0.86,1.44]	1.24 [0.96,1.59]	1.42 [1.17,1.73]***	1.34 [0.75,2.41]
30m	1.57 [1.20,2.07]***	1.52 [1.14,2.03]**	1.48 [1.10, 1.99]**	1.78 [1.41,2.25]***	3.40 [2.06,5.64]***
42m	1.33 [0.93,1.90]	1.92 [1.38,2.69]***	1.74 [1.24,2.46]***	1.52 [1.14,2.03]**	3.77 [2.11,6.74]***
Ears turn red					
18m	1.19 [1.01,1.40]*	1.09 [0.92,1.29]	1.13 [0.95,1.34]	1.46 [1.28,1.66]***	1.10 [0.72,1.69]
30m	1.35 [1.13,1.61]***	1.17 [0.96,1.41]	1.20 [0.99,1.45]	1.54 [1.33,1.79]***	2.14 [1.44,3.17]***
42m	1.27 [1.05,1.53]*	1.21 [0.99,1.47]	1.05 [0.86,1.30]	1.33 [1.14,1.56]***	1.52 [0.98,2.37]
Pus/mucus from ears					
18m	0.98 [0.65,1.48]	1.27 [0.86,1.90]	1.26 [0.86,1.85]	1.14 [0.83,1.57]	1.87 [0.85,4.08]
30m	1.12 [0.78,1.60]	1.32 [0.92,1.89]	1.19 [0.83,1.71]	1.33 [1.00,1.75]*	3.29 [1.85,5.86]***
42m	1.31 [1.01,1.72]*	1.23 [0.93,1.64]	1.22 [0.91,1.65]	1.31 [1.05,1.64]*	2.85 [1.72,4.72]***
Hearing worse during a cold					
18m	1.22 [0.88,1.71]	1.53 [1.10,2.14]*	1.22 [0.86,1.73]	1.25 [0.95,1.64]	0.82 [0.33,2.06]
30m	1.33 [1.10,1.62]**	1.35 [1.11,1.65]**	1.22 [1.00,1.49]	1.40 [1.20,1.64]***	2.18 [1.43,3.31]***
42m	1.47 [1.20,1.79]***	1.73 [1.41,2.12]***	1.10 [0.88,1.37]	1.59 [1.35,1.88]***	3.28 [2.15,5.00]***
Rarely listens to nearby sounds					
18m	2.40 [1.61,3.58]***	1.58 [1.00,2.51]	2.07 [1.40,3.06]***	1.89 [1.32,2.72]***	2.26 [0.97,5.27]
30m	2.56 [1.76,3.73]***	2.69 [1.82,3.97]***	2.87 [2.01,4.10]***	2.57 [1.84,3.59]***	6.44 [3.68,11.28]***
42m	2.64 [1.96,3.57]***	2.76 [2.01,3.79]***	2.31 [1.70,3.15]***	2.11 [1.60,2.79]***	6.60 [4.04,10.77]***

*P< 0.05; ** P < 0.01; *** P < 0.001; AOR = Adjusted odds ratio

Differences in breathing

For peer review only

The unadjusted comparisons showed that mouth breathing (all of the time or much of the time) was positively associated with autistic traits and autism at each of the three ages at which the question was asked, with P<0.05 for 14 of the 15 data points. Similarly, snoring at each of the three time points showed positive associations with the autism outcomes, all of the 15 comparisons being at P<0.05. However, for symptoms of sleep apnoea (often or sometimes), although there were always more affected children with the autistic outcome, only 10 of 15 associations showed differences at P<0.05 (Supplementary Tables 1-3).

On adjustment (Table 4) the three signs in this category showed different associations: social communication and coherence showed associations with all three signs, repetitive behaviour with two of the signs (mouth breathing and sleep apnoea) but sociability only with mouth breathing and only at one time point (18 months). Although numbers with autism were small, the adjusted odds ratio (AOR) was as high as 1.93 and 2.13 for mouth breathing and snoring respectively.

Signs of ear differences

The proportion of children who often pulled, scratched, or poked at their ears also showed positive unadjusted associations at P<0.05 in 11 of the 15 autism outcomes, and 14 of 15 associations for a history of the child's ears being "red and sore looking". "Pus leaking from the ear (more than once)" was associated with lower number of autistic outcomes (7 of 15), with particularly strong associations with speech coherence and autism (Supplementary Tables 4-6).

After adjustment (Table 4), pulling, scratching, or poking at ears was particularly strongly associated with autism, with AORs of 3.40 (95%CI 2.06, 5.640 and 3.77 (95%CI 2.11, 6.74) at 30 and 42 months. There were also strong associations for each autistic trait: the age at maximum odds varied from 30 months for social communication and repetitive behaviour, to 42 months for speech coherence and sociability. The adjusted odds were less marked for children whose ears were reported to have gone red for prolonged periods of time and were only associated with two traits (social communication and repetitive behaviour). For autism the association was strongest at 30m with an AOR of 2.14 (95% CI 1.44, 3.17). For pus from the ears, there were only mild associations with the autistic traits, but strong associations with autism, particularly at 30 months with an AOR of 3.29. (95%CI 1.85, 5.86).

Signs of listening and hearing difficulties

On unadjusted analyses, reports of the child's hearing being worse during or after a cold were associated with autism and for three of the four autistic traits (sociability being the exception). The differences of the 4 outcomes were apparent at each of the three ages at P<0.0001 (Supplementary Table 7). The unadjusted pattern was different in relation to the question concerning whether the child listens to people or to things that happen nearby. Here all comparisons were at P<0.05, including the poor sociability outcome (Supplementary Tables 7 and 8).

On adjustment, the results are similar to the unadjusted associations (Table 4), with the AORs being particularly high for failure to react to noise nearby at 30 months of age for children with autism [AOR = 6.44, 95% CI 3.68, 11.28; P<0.0001].

Discussion

For peer review only - http://bmjopen.bmj.com/site/about/guidelines.xhtml

There is increasing interest world-wide in autism, and much research has been undertaken to understand the neuro-cognitive and genetic differences.[36] In this set of analyses we have shown that common indicators of ear health and upper respiratory compromise are more frequent in children subsequently identified with autism as well as in children who have high levels of several autistic traits. These associations may be important because either (a) that these ear and respiratory signs are early markers of increased risk of autism; or that (b) they may inform the origins of autism, or (c) that they may highlight co-occurring conditions which if treated may lead to a better quality of life for children with autism.

No birth cohort studies, to our knowledge, have investigated prospectively the common signs that might indicate upper respiratory disadvantage such as snoring, mouth breathing or sleep apnoea as precursors of autism. Sleep problems are commonly reported by parents of children with autism, including problems due to sleep-related breathing disorders, such as snoring or apnoea.[37] In this study we found that mouth breathing in the first three years of life was associated with autism, but not symptoms of sleep apnoea, or early snoring except at 42 months. However, both snoring and symptoms of sleep apnoea in the early years were linked to the autistic traits of social communication and speech coherence, across most of the time points; sleep apnoea was also linked to repetitive behaviour. Studies examining whether sleep related breathing disorders are more commonly reported in autism than in typically developing children are generally case-control studies of children with a confirmed diagnosis of autism. Results generally show more sleep disordered breathing in children with autism,[10 11 38 39 40] although Alfonso-Alfonso et al.[41] found no difference and Malow and colleagues[42] carried out polysomnography in children with autism and typically developing controls and found no physiological evidence of sleep apnoea in the autism group. Our findings based on a large sample size may indicate that children who develop autistic traits have a slightly disordered upper respiratory structure or physiology, such as obstructed airways from enlarged adenoids. For example, snoring results during sleep when the upper respiratory system relaxes and various organs in the airways vibrate and breathing through the mouth rather than the nose is an indicator of obstructed airways, as is breath holding or stopping breathing during sleep.

Similarly, subtle signs of possible middle ear disease such as pulling or poking at the ears, the ears going red for a prolonged period of time, or pus or sticky mucus discharging from the ears appear not to have been considered before in birth cohort studies. We found that these signs were closely associated with autistic traits and with a diagnosis of autism, especially if the signs were present at around 30 months of age. There were also strong relationships between ear discharge and autism. Ear infections have been linked to autism based on medical attendance for otitis media in health registry studies,[4 7 8] through prospective parental report of ear infections in a birth cohort study[19] and retrospective parental report in a case-control study.[14] Interestingly, a study based on the Danish National Birth Cohort found no association between parental report of ear infections and autism, but did find an association when hospital contact for ear infections was examined as the exposure[43]; the authors suggest the hospital record data could be affected by both detection and selection bias which is less likely to influence studies involving prospective parental reporting. In order to determine whether the child's hearing was affected in the absence of direct hearing tests, we assessed the responses to questions concerning whether the child's hearing was thought by the mother to be worse during a cold, and whether she reported that the child rarely listened to people or things that happened nearby. Both characteristics were associated with autism and the autistic traits, particularly at 30 months. Although unresponsive behaviour is a feature of the social communication autistic trait, the associations

For peer review only - http://bmjopen.bmj.com/site/about/guidelines.xhtml

with listening difficulties accompanying a cold implies the presence of a fluctuating conductive hearing loss rather than inattention. This finding is consistent with case-control studies that have shown higher levels of abnormal tympanograms (middle ear function tests indicating the presence of otitis media) in autism.[15 44 45] There is limited evidence however that older children with autism have peripheral hearing loss, with relevant studies systematically reviewed in 2014.[46]

This study adds to the evidence that, compared to a typical population of the same age, early ear and upper respiratory symptoms are more common in those subsequently diagnosed with autism or with extreme levels of autistic traits. It is not possible to determine whether these ENT conditions have a causal role in the development of autistic traits or are related to an unmeasured factor. One possibility, for example, could be the consequence of the increased prevalence of minor physical anomalies in individuals with autism,[47] including anatomical differences in the structure and/or positioning of the ear[14 48] with such differences in ear morphology increasing the risk of ENT conditions.

Ongoing otitis media with effusion during early childhood leads to impairment in auditory processing, although this improves as otitis media clears.[49] Atypical sensory processing is a common presentation in children with autism,[50 51] but also the prevalence of autism is higher in deaf children than hearing children and similarly higher in children with visual impairment.[52] This suggests that in children with an existing susceptibility to development of autism, disrupted auditory processing as a result of otitis media may interact with existing deficiencies in sensory processing and integration, impacting on development of autistic traits.[50]

Aside from suggestions as to mechanisms, it is clear from this study of prospectively collected information, that children who later develop social communication difficulties are more likely to have early middle ear disease and ENT conditions and are therefore more at risk of communication difficulties from hearing loss, albeit temporary. Early detection and intervention of ENT conditions in children with autism is thus likely to be beneficial.

Strengths and Limitations

The strength of the study lies in the fact that the population is geographically defined, and includes the great majority (~80%) of the eligible population. The data are possibly unique in documenting common early ear and upper respiratory signs and symptoms of the children who subsequently develop extreme levels of autistic traits and/or a diagnosis of autism. The mothers answered structured questions at distinct time points with no idea as to whether the signs they were describing were linked to outcomes such as autism. Thus, there were no discernible biases in ascertainment of these signs, or of the later identification of the autistic traits.

The limitations, as in all longitudinal studies, lie in the loss of children to later follow up.[21] Unfortunately, at the time the children were born there were very few families of ethnic minorities resident in the area (~6%). Consequently, our results cannot be extrapolated to cover non-white populations in general. A further limitation concerns the fact that the study children were not examined consistently to determine a diagnosis of autism. Rather a strategy to assess the probability of a diagnosis using a variety of different sources was used. The validity of this approach was shown since the group of children identified in this way demonstrates a positive correlation with a polygenic risk score for autism.

For peer review only

It is always possible that the statistical analyses did not allow for all the appropriate confounders, and further data sets are needed to assess the validity of our results.

Conclusion

Data collected longitudinally from 18 to 42 months has demonstrated close associations of common early ear and upper respiratory markers with a later diagnosis of autism as well as with high levels of autistic traits. These markers included snoring and mouth breathing as well as a reduction in hearing ability during a cold, and signs of otitis media with infusion. The data indicate that the strongest associations occurred at age 30 months. These results may indicate disruptions in early auditory perception and processing in the early stages of autism.

Data availability

ALSPAC data is available to researchers for particular projects, provided no attempt is made to reveal the identities of the subjects. Guidelines for access are found on the ALSPAC website: *www.bristol.ac.uk/alspac/researchers*

Ethics

Ethical approval for the study was obtained from the ALSPAC Ethics and Law Committee (ALEC; IRB00003312) and the Local Research Ethics Committees. Detailed information on the ways in which confidentiality of the cohort is maintained may be found on the study website: http://www.bristol.ac.uk/alspac/researchers/research-ethics/

All methods were performed in accordance with the relevant guidelines and regulations. Informed consent for the use of data collected via questionnaires and clinics was obtained from participants following the recommendations of the ALSPAC Ethics and Law Committee at the time.[23]

Acknowledgements

We are extremely grateful to all the families who took part in this study, the midwives for their help in recruiting them, and the whole ALSPAC team, which includes interviewers, computer and laboratory technicians, clerical workers, research scientists, volunteers, managers, receptionists, and nurses.

Contributors:

Conception and design of study: AH and JG
Acquisition of funding: JG and YIC
Preparation of study data and Analyses: AH and SG
Statistical analysis quality checking: JG and AH
Original draft: AH and JG
All authors contributed to interpretation of the findings.
All authors contributed to the critical review and editing of the manuscript and approved the final manuscript.
All authors are guarantors for this work.
The corresponding author attests that all listed authors meet authorship criteria and that no others meeting the criteria have been omitted.

For peer review only

Funding

This publication was made possible through the support of grants from the Medical Research Council (G0400085) and the John Templeton Foundation (ref no. 61917). The UK Medical Research Council and Wellcome Trust (Grant ref: 217065/Z/19/Z) and the University of Bristol (grant ref: N/A) currently provide core support for ALSPAC.

This publication is the work of the authors who will serve as guarantors for the contents of this paper. A comprehensive list of grants funding is available on the ALSPAC website (http://www.bristol.ac.uk/alspac/external/documents/grant-acknowledgements.pdf). The opinions expressed in this publication are those of the author(s) and do not necessarily reflect the views of the John Templeton Foundation.

Competing interests: JG and RM are retired; YIC and SG are funded by *the John Templeton Foundation (*61917); no financial relationships with any organisations that might have an interest in the submitted work in the previous three years; no other relationships or activities that could appear to have influenced the submitted work.

Patient Participation & Involvement

Patients and/or the public were not involved in the reporting or dissemination plans of this research. Prior to data collection (in the early 1990s), focus groups of pregnant women were involved in discussions on the content of questionnaires.

Provenance & Peer Review

Not commissioned; externally peer reviewed.

For peer review only

References

1. Lai MC, Lombardo MV, Baron-Cohen S. Autism. *Lancet* 2014;**383**:896-910.
2. Happe F, Ronald A, Plomin R. Time to give up on a single explanation for autism. *Nat Neurosci,* 2006;**9**:1218-20.
3. Tye C, Runicles AK, Whitehouse AJO, et al. Characterizing the interplay between autism spectrum disorder and comorbid medical conditions: an integrative review. *Front Psychiatry* 2018;**9**:751
4. Doshi-Velez F, Ge Y, Kohane I. Comorbidity clusters in autism spectrum disorders: an electronic health record time-series analysis. *Pediatrics* 2014;**133**:e54-63.
5. Alexeeff SE, Yau V, Qian Y, et al. Medical conditions in the first years of life associated with future diagnosis of asd in children. *J Autism Dev Disord* 2017;**47**:2067-79.
6. Gurney JG, McPheeters ML, Davis MM. Parental report of health conditions and health care use among children with and without autism: national survey of children's health. *Arch Pediatr Adolesc Med* 2006;**160**:825-
7. Adams DJ, Susi A, Erdie-Lalena CR, et al. Otitis media and related complications among children with autism spectrum disorders. *J Autism Dev Disord* 2016; **46**:1636-42.
8. Cawthorpe D. A 16-year cohort analysis of autism spectrum disorder-associated morbidity in a pediatric population. *Front Psychiatry* 2018;**9**:635.
9. Rosen NJ, Yoshida CK, Croen LA. Infection in the first 2 years of life and autism spectrum disorders. *Pediatrics* 2017;**119**:e61-9.
10. Hodge D, Carollo TM, Lewin M, et al. Sleep patterns in children with and without autism spectrum disorders: developmental comparisons. *Res Dev Disabil* 2014;**35**:1631-8.
11. Hirata I, Mohri I, Kato-Nishimura K, et al. Sleep problems are more frequent and associated with problematic behaviors in preschoolers with autism spectrum disorder. *Res Dev Disabil,* 2016;**49-50**:86-99.
12. Elrod MG, Nylund CM, Susi AL, et al. Prevalence of diagnosed sleep disorders and related diagnostic and surgical procedures in children with autism spectrum disorders. *J Dev Behav Pediatr* 2016;**37**: 377-84
13. Ackerman S, Reilly B, Bernier R. Tympanostomy tube placement in children with autism. *J Dev Behav Pediatr* 2012;**33**:252-8.
14. Konstantareas MM, Homatidis S. Ear infections in autistic and normal children. *J Autism Dev Disord* 1987;**17**:585-94.
15. Rafal Z. Conductive hearing loss in children with autism. *Eur J Pediatr* 2013;**172**: 1007-10.
16. Demopoulos C, Lewine JD. Audiometric profiles in autism spectrum disorders: does subclinical hearing loss impact communication? *Autism Res* 2016;**9**:107-20.
17. Gravel JS, Dunn M, Lee WW, et al. Peripheral audition of children on the autistic spectrum. *Ear Hear* 2016;**27**:299-312.
18. Tharpe AM, Bess FH, Sladen DP, et al. Auditory characteristics of children with autism. *Ear Hear* 2006;**27**:430-41.
19. Jeans LM, Santos RM, Laxman DJ, et al. Early predictors of asd in young children using a nationally representative data set. *J Early Interven* 2013;**35**:303-31.
20. Golding J, Pembrey M, Jones R. ALSPAC--the avon longitudinal study of parents and children. I. Study methodology. *Paediatr Perinat Epidemiol* 2001;**15**:74-87.
21. Boyd A, Golding J, Macleod J, et al. Cohort profile: the 'children of the 90s'--the index offspring of the avon longitudinal study of parents and children. *Int J Epidemiol* 2013;42:111-27.

For peer review only

22. Fraser A, Macdonald-Wallis C, Tilling K, et al. Cohort Profile: The Avon Longitudinal Study Of Parents And Children: ALSPAC mothers cohort. *Int J Epidemiol* 2013;**42**:97-110.
23. Birmingham K. Pioneering ethics in longitudinal studies: The early development of the ALSPAC Ethics & Law Committee. Policy Press, 2018.
24. Williams E, Thomas K, Sidebotham H, et al. Prevalence and characteristics of autistic spectrum disorders in the ALSPAC cohort. *Dev Med Child Neurol* 2008; **50**:672-7.
25. Goodman R, Ford T, Richards H, et al. The development and well-being assessment: description and initial validation of an integrated assessment of child and adolescent psychopathology. *J Child Psychol Psychiatry* 2000;**41**:645-55.
26. Rai D, Culpin I, Heuvelman H, et al. Association of autistic traits with depression from childhood to age 18 years. *JAMA Psychiatry* 2018;**75**:835-43.
27. Steer CD, Golding J, Bolton PF. Traits contributing to the autistic spectrum. *Plos One* 2010;**5**:e12633.
28. Skuse DH, Mandy WP, Scourfield J. Measuring autistic traits: heritability, reliability and validity of the Social And Communication Disorders Checklist. *Br J Psychiatry,* 2005;**187**:568-72.
29. Skuse DH, Mandy W, Steer C, et al. Social communication competence and functional adaptation in a general population of children: preliminary evidence for sex-by-verbal IQ differential risk. *J Am Acad Child Adolesc Psychiatry* 2009;**48**:128-37.
30. St Pourcain B, Mandy WP, Heron J, et al. Links between co-occurring social-communication and hyperactive-inattentive trait trajectories. *J Am Acad Child Adolesc Psychiatry* 2011;**50**:892-902.e5
31. Bishop DV. Development of the children's communication checklist (ccc): a method for assessing qualitative aspects of communicative impairment in children. *J Child Psychol Psychiatry* 1998;**39**:879-91.
32. Bishop DV, Baird G. Parent and teacher report of pragmatic aspects of communication: use of the children's communication checklist in a clinical setting. *Dev Med Child Neurol* 2001;**43**:809-18.
33. Buss AH, Plomin R. Temperament: early developing personality traits. L Erlbaum Associates, 1984.
34. Cox JL, Holden JM, Sagovsky R. Detection of postnatal depression. Development of the 10-item Edinburgh Postnatal Depression Scale. *Br J Psychiatry* 1987;**150**:782-6.
35. Golding J, Gregory S, Iles-Caven Y, et al. The antecedents of women's external locus of control: Associations with characteristics of their parents and their early childhood. *Heliyon* 2017;**3**:e00236.
36. Lyall K, Croen L, Daniels J, et al. The changing epidemiology of autism spectrum disorders. *Ann Rev Public Health* 2017;**38**:81-102.
37. Schreck KA, Richdale AL. Sleep problems, behavior, and psychopathology in autism: inter-relationships across the lifespan. *Curr Opin Psychol* 2020;**34**:105-11.
38. Schreck KA, Mulick JA. Parental report of sleep problems in children with autism. *J Autism Dev Disord* 2000;**30**:127-35.
39. Trickett J, Heald M, Oliver C, et al. A cross-syndrome cohort comparison of sleep disturbance in children with Smith-Magenis syndrome, Angelman syndrome, autism spectrum disorder and tuberous sclerosis complex. *J Neurodev Disord* 2018;**10**:9.
40. Mutluer T, Karakoc Demirkaya S, Abali O. Assessment of sleep problems and related risk factors observed in Turkish children with Autism spectrum disorders. *Autism Res* 2016;**9**:536-42.

For peer review only

41. Alfonso-Alfonso M, Morales-Chacón LM, González-Naranjo JE. Subjective assessment of sleep in infantile autism: a comparative study. *Behav Sci (Basel)* 2019;**9**
42. Malow BA, Marzec ML, McGrew SG, et al. Characterizing sleep in children with autism spectrum disorders: a multidimensional approach. *Sleep* 2006;**29**:1563-71.
43. Atladottir HO, Henriksen TB, Schendel DE, et al. Using maternally reported data to investigate the association between early childhood infection and autism spectrum disorder: the importance of data source. *Paediatr Perinat Epidemiol* 2012;**26**:373-85.
44. Rosenhall U, Nordin V, Sandstrom M, et al. Autism and hearing loss. *J Autism Dev Disord* 1999;**29**:349-57.
45. Smith DE, Miller SD, Stewart M, et al. Conductive hearing loss in autistic, learning-disabled, and normal children. *J Autism Dev Disord* 1988;**18**:53-65.
46. Beers AN, McBoyle M, Kakande E, et al. Autism and peripheral hearing loss: a systematic review. *Int J Pediatr Otorhinolaryngol* 2014;**78**: 96-101.
47. Ozgen HM, Hop JW, Hox JJ, et al. Minor physical anomalies in autism: a meta-analysis. *Mol Psychiatry* 2010;**15**:300-7.
48. Rodier PM, Bryson SE, et al. Minor malformations and physical measurements in autism: data from Nova Scotia. *Teratol* 1997;**55**:319-25.
49. Moore DR, Hartley DE, Hogan SC. Effects of otitis media with effusion (OME) on central auditory function. *Int J Pediatr Otorhinolaryngol* 2003;**67**:S63-7.
50. Thye MD, Bednarz HM, Herringshaw AJ, et al. The impact of atypical sensory processing on social impairments in autism spectrum disorder. *Dev Cogn Neurosci* 2018; **29**:151-67.
51. O'Connor K. Auditory processing in autism spectrum disorder: a review. *Neurosci Biobehav Rev* 2012;**36**:836-54.
52. Do B, Lynch P, Macris EM, et al. Systematic review and meta-analysis of the association of Autism Spectrum Disorder in visually or hearing impaired children. *Ophthalmic Physiol Opt* 2017;**37**:212-24.

For peer review only

Hearing and autism: Supplementary Tables

Supplementary Table 1. Comparison of the prevalence of **mouth breathing** among index children (i.e. with high levels of autistic traits or diagnosed autism) and control children (i.e. all who were not in the index category)

**Age**		**SCDC % (n)**	**Coherence % (n)**	**Sociability % (n)**	**Repetitive % (n)**	**Autism % (n)**
18m	Index	13.1 (117)	15.1 (99)	15.1 (143)	13.7 (289)	17.4 (24)
	Control	10.2 (627)	10.5 (633)	11.4 (875)	9.9 (540)	12.2 (1196)
	P	**	****	****	****	*
30m	Index	27.2 (232)	26.5 (169)	24.5 (226)	24.1 (489)	30.3 (42)
	Control	19.1 (1119)	19.8 (1145)	20.8 (1524)	19.0 (992)	21.8 (1966)
	P	****	****	*	****	**
42m	Index	26.9 (260)	25.4 (185)	23.3 (250)	24.9 (573)	25.5 (38)
	Control	18.6 (1219)	18.9 (1228)	20.3 (1690)	18.8 (1013)	20.7 (2054)
	P	****	****	*	****	-

*P< 0.05; ** P < 0.01; *** P < 0.001; **** P < 0.0001

Supplementary Table 2. Comparison of the prevalence of **snoring** among index children (i.e. with high levels of autistic traits or diagnosed autism) and control children (i.e. all who were not in the index category)

**Age**		**SCDC % (n)**	**Coherence % (n)**	**Sociability % (n)**	**Repetitive % (n)**	**Autism % (n)**
18m	Index	15.3 (144)	14.6 (102)	11.8 (122)	11.5 (258)	16.3 (24)
	Control	7.6 (495)	7.9 (511)	8.8 (719)	7.3 (427)	9.5 (998)
	P	****	****	**	****	*
30m	Index	25.4 (221)	22.0 (150)	20.9 (196)	20.2 (417)	31.0 (41)
	Control	16.0 (947)	16.6 (967)	17.4 (1283)	16.1(851)	18.2 (1660)
	P	****	****	**	****	***
42m	Index	24.6 (238)	23.2 (169)	22.2 (237)	22.0 (506)	26.2 (39)
	Control	17.6 (1152)	17.9 (1158)	18.5 (1534)	17.3 (1020)	19.1 (1889)
	P	****	****	**	****	*

*P< 0.05; ** P < 0.01; *** P < 0.001; **** P < 0.0001

Supplementary Table 3. Comparison of the prevalence of possible **sleep apnoea** among index children (i.e. with high levels of autistic traits or diagnosed autism) and control children (i.e. all who were not in the index category).

Age		SCDC % (n)	Coherence % (n)	Sociability % (n)	Repetitive % (n)	Autism % (n)
18m	Index	10.8 (91)	8.8 (54)	8.2 (77)	9.3 (189)	9.9 (13)
	Control	6.8 (405)	7.0 (409)	7.9 (590)	6.9 (367)	7.9 (769)
	P	****	*	-	**	-
30m	Index	18.1 (148)	18.4 (110)	15.6 (143)	17.4 (339)	16.8 (21)
	Control	14.0 (802)	13.9 (789)	15.2 (1099)	13.6 (705)	15.5 (1387)
	P	***	***	-	****	-
42m	Index	16.4 (158)	17.4 (126)	13.8 (148)	15.4 (355)	18.8 (28)
	Control	12.2 (799)	12.1 (784)	12.9 (1073)	12.0 (709)	14.2 (1298)
	P	***	****	-	****	*

*P< 0.05; ** P < 0.01; *** P < 0.001; **** P < 0.0001

Supplementary Table 4. Comparison of the prevalence of **often pulling and poking at ears** among index children (i.e. with high levels of autistic traits or diagnosed autism) and control children (i.e. all who were not in the index category).

Age		SCDC % (n)	Coherence % (n)	Sociability % (n)	Repetitive % (n)	Autism % (n)
18m	Index	10.4 (103)	10.6 (77)	9.3 (99)	9.8 (226)	10.9 (17)
	Control	6.9 (460)	6.9 (456)	7.5 (634)	6.6 (395)	7.9 (862)
	P	****	****	*	****	-
30m	Index	8.2 (78)	8.4 (60)	6.8 (71)	7.0 (157)	16.2 (25)
	Control	4.1 (267)	4.2 (268)	4.4 (353)	3.7 (213)	4.6 (463)
	P	****	****	**	****	****
42m	Index	1.8 (17)	2.8 (20)	2.2 (24)	2.5 (58)	10.7 (16)
	Control	1.8 (116)	1.8 (115)	2.0 (163)	1.8 (106)	5.3 (521)
	P	-	-	-	*	***

*P< 0.05; ** P < 0.01; *** P < 0.001; **** P < 0.0001

For peer review only

Supplementary Table 5. Comparison of the prevalence of **ears going red and sore looking for a long time** among index children (i.e. with high levels of autistic traits or diagnosed autism) and control children (i.e. all who were not in the index category).

**Age**		**SCDC**	**Coherence**	**Sociability**	**Repetitive**	**Autism**
18m	Index	27.3 (261)	27.4 (194)	24.9 (259)	26.6 (602)	25.3 (38)
	Control	19.6 (1290)	19.9 (1292)	21.0 (1736)	18.3 (1072)	21.6 (2298)
	P	****	****	**	****	-
30m	Index	21.0 (221)	20.4 (139)	18.0 (181)	19.2 (418)	26.5 (40)
	Control	13.0 (820)	13.5 (841)	14.6 (1150)	12.1(680)	15.0 (1458)
	P	****	****	**	****	***
42m	Index	17.0 (164)	17.7 (129)	14.7 (158)	16.1 (371)	19.5 (29)
	Control	12.1 (792)	12.0 (779)	12.9 (14)	11.3 (670)	13.2 (1303)
	P	****	****	**	****	*

*P< 0.05; ** P < 0.01; *** P < 0.001; **** P < 0.0001

Supplementary Table 6. Comparison of the prevalence of **pus/sticky mucus leaking from ears more than once** among index children (i.e. with high levels of autistic traits or diagnosed autism) and control children (i.e. all who were not in the index category).

**Age**		**SCDC % (n)**	**Coherence % (n)**	**Sociability % (n)**	**Repetitive % (n)**	**Autism % (n)**
18m	Index	2.8 (27)	4.7 (34)	3.8 (40)	3.2 (73)	5.3 (8)
	Control	2.8 (184)	2.6 (169)	3.0 (250)	2.7 (159)	3.0 (322)
	P	-	***	-	-	-
30m	Index	3.9 (37)	5.1 (36)	4.4 (46)	4.0 (90)	10.3 (16)
	Control	3.2 (207)	3.0 (189)	3.3 (263)	3.1(177)	6.6 (349)
	P	-	**	-	-	***
42m	Index	8.3 (80)	10.3 (75)	7.2 (77)	7.3 (167)	15.4 (23)
	Control	5.2 (342)	5.2 (339)	5.7 (471)	5.3 (311)	5.8 (575)
	P	****	****	-	****	****
3-4 y	Index	4.8 (44)	4.7 (33)	5.6 (55)	4.0 (90)	9.6 (14)
	Control	3.2 (207)	3.2 (200)	3.2 (245)	3.1 (181)	3.5 (324)
	P	**	**	**	-	**

*P< 0.05; ** P < 0.01; *** P < 0.001; **** P < 0.0001

For peer review only

Supplementary Table 7. Comparison of the prevalence of **hearing reported to be worse during a cold** among index children (i.e. with high levels of autistic traits or diagnosed autism) and control children (i.e. all who were not in the index category).

**Age**		**SCDC % (n)**	**Coherence % (n)**	**Sociability % (n)**	**Repetitive % (n)**	**Autism % (n)**
18m	Index	20.2 (173)	21.9 (137)	17.7 (165)	18.5 (369)	29.9 (41)
	Control	14.4 (842)	14.6 (844)	15.4 (1143)	13.9 (736)	15.4 (1485)
	P	****	****	-	****	****
30m	Index	21.5 (148)	25.8 (133)	16.5 (127)	20.1 (319)	37.9 (44)
	Control	14.1 (682)	14.1 (673)	15.6 (946)	13.1(574)	15.2 (1136)
	P	****	****	-	****	****
42m	Index	37.1 (354)	41.4 (297)	31.6 (335)	36.3 (827)	52.4 (77)
	Control	29.9 (1943)	29.3 (1880)	30.5 (2512)	28.6 (1676)	30.5 (2989)
	P	****	****	-	****	****

*P< 0.05; ** P < 0.01; *** P < 0.001; **** P < 0.0001

Supplementary Table 8. Comparison of the prevalence of **rarely listening** among index children (i.e. with high levels of autistic traits or diagnosed autism) and control children (i.e. all who were not in the index category).

**Age**		**SCDC % (n)**	**Coherence % (n)**	**Sociability % (n)**	**Repetitive % (n)**	**Autism % (n)**
18m	Index	4.2 (42)	3.9 (29)	3.8 (40)	3.0 (70)	5.1 (8)
	Control	1.6 (108)	1.6 (103)	1.9 (162)	1.7 (98)	2.1 (235)
	P	****	****	*	*	*
30m	Index	4.6 (44)	6.4 (45)	5.1 (53)	4.1 (91)	14.4 (22)
	Control	1.6 (101)	1.5 (95)	1.7 (139)	1.4 (80)	2.1 (213)
	P	****	****	****	****	****
42m	Index	7.7 (75)	8.9 (65)	6.7 (72)	5.5 (126)	20.2 (30)
	Control	2.5 (165)	2.3 (148)	2.8 (235)	2.3 (135)	3.1 (306)
	P	****	****	****	****	****

*P< 0.05; ** P < 0.01; *** P < 0.001; **** P < 0.0001

For peer review only

STROBE Statement—Checklist of items that should be included in reports of ***cohort studies***

	Item No	Recommendation	Page No
**Title and abstract**	1	(*a*) Indicate the study's design with a commonly used term in the title or the abstract	1
		(*b*) Provide in the abstract an informative and balanced summary of what was done and what was found	2
**Introduction**			
Background/rationale	2	Explain the scientific background and rationale for the investigation being reported	4
Objectives	3	State specific objectives, including any prespecified hypotheses	5
**Methods**			
Study design	4	Present key elements of study design early in the paper	5
Setting	5	Describe the setting, locations, and relevant dates, including periods of recruitment, exposure, follow-up, and data collection	5
Participants	6	(*a*) Give the eligibility criteria, and the sources and methods of selection of participants. Describe methods of follow-up	6
		(*b*) For matched studies, give matching criteria and number of exposed and unexposed	
Variables	7	Clearly define all outcomes, exposures, predictors, potential confounders, and effect modifiers. Give diagnostic criteria, if applicable	6-8
Data sources/ measurement	8*	For each variable of interest, give sources of data and details of methods of assessment (measurement). Describe comparability of assessment methods if there is more than one group	6-8
Bias	9	Describe any efforts to address potential sources of bias	8-9
Study size	10	Explain how the study size was arrived at	6
Quantitative variables	11	Explain how quantitative variables were handled in the analyses. If applicable, describe which groupings were chosen and why	6-9
Statistical methods	12	(*a*) Describe all statistical methods, including those used to control for confounding	9
		(*b*) Describe any methods used to examine subgroups and interactions	N/A
		(*c*) Explain how missing data were addressed	
		(*d*) If applicable, explain how loss to follow-up was addressed	
		(*e*) Describe any sensitivity analyses	
**Results**			
Participants	13*	(a) Report numbers of individuals at each stage of study—eg numbers potentially eligible, examined for eligibility, confirmed eligible, included in the study, completing follow-up, and analysed	6,13
		(b) Give reasons for non-participation at each stage	N/A
		(c) Consider use of a flow diagram	N/A
Descriptive data	14*	(a) Give characteristics of study participants (eg demographic, clinical, social) and information on exposures and potential confounders	20
		(b) Indicate number of participants with missing data for each variable of interest	
		(c) Summarise follow-up time (eg, average and total amount)	N/A
Outcome data	15*	Report numbers of outcome events or summary measures over time	6-8

For peer review only - http://bmjopen.bmj.com/site/about/guidelines.xhtml

Main results	16	(*a*) Give unadjusted estimates and, if applicable, confounder-adjusted estimates and their precision (eg, 95% confidence interval). Make clear which confounders were adjusted for and why they were included	9-11
		(*b*) Report category boundaries when continuous variables were categorized	20
		(*c*) If relevant, consider translating estimates of relative risk into absolute risk for a meaningful time period	
Other analyses	17	Report other analyses done—eg analyses of subgroups and interactions, and sensitivity analyses	N/A
**Discussion**			
Key results	18	Summarise key results with reference to study objectives	11-13
Limitations	19	Discuss limitations of the study, taking into account sources of potential bias or imprecision. Discuss both direction and magnitude of any potential bias	13
Interpretation	20	Give a cautious overall interpretation of results considering objectives, limitations, multiplicity of analyses, results from similar studies, and other relevant evidence	11-13
Generalisability	21	Discuss the generalisability (external validity) of the study results	14
**Other information**			
Funding	22	Give the source of funding and the role of the funders for the present study and, if applicable, for the original study on which the present article is based	14

*Give information separately for exposed and unexposed groups.

Note: An Explanation and Elaboration article discusses each checklist item and gives methodological background and published examples of transparent reporting. The STROBE checklist is best used in conjunction with this article (freely available on the Web sites of PLoS Medicine at http://www.plosmedicine.org/, Annals of Internal Medicine at http://www.annals.org/, and Epidemiology at http://www.epidem.com/). Information on the STROBE Initiative is available at http://www.strobe-statement.org.

For peer review only - http://bmjopen.bmj.com/site/about/guidelines.xhtml
For peer review only

BMJ Open

Associations between autistic traits and early ear and upper respiratory signs: a prospective observational study of the ALSPAC geographically defined childhood population

Journal:	*BMJ Open*
Manuscript ID	bmjopen-2022-067682.R1
Article Type:	Original research
Date Submitted by the Author:	31-Jan-2023
Complete List of Authors:	Hall, Amanda; Aston University, Life and Health Sciences<br>Maw, Richard; University of Bristol Faculty of Medicine and Dentistry, St Michael's Hospital<br>Iles-Caven, Yasmin; University of Bristol Faculty of Medicine and Dentistry, Bristol Medical School (PHS)<br>Gregory, Steven; University of Bristol Faculty of Medicine and Dentistry, Bristol Medical School (PHS)<br>Rai, Dheeraj; University of Bristol Faculty of Medicine and Dentistry, Centre for Academic Mental Health<br>Golding, Jean; University of Bristol Faculty of Medicine and Dentistry, Bristol Medical School (PHS)
**Primary Subject Heading**:	Ear, nose and throat/otolaryngology
Secondary Subject Heading:	Paediatrics, Epidemiology
Keywords:	Paediatric otolaryngology < OTOLARYNGOLOGY, EPIDEMIOLOGY, Audiology < OTOLARYNGOLOGY, Delirium & cognitive disorders < PSYCHIATRY

SCHOLARONE™
Manuscripts

I, the Submitting Author has the right to grant and does grant on behalf of all authors of the Work (as defined in the below author licence), an exclusive licence and/or a non-exclusive licence for contributions from authors who are: i) UK Crown employees; ii) where BMJ has agreed a CC-BY licence shall apply, and/or iii) in accordance with the terms applicable for US Federal Government officers or employees acting as part of their official duties; on a worldwide, perpetual, irrevocable, royalty-free basis to BMJ Publishing Group Ltd ("BMJ") its licensees and where the relevant Journal is co-owned by BMJ to the co-owners of the Journal, to publish the Work in this journal and any other BMJ products and to exploit all rights, as set out in our licence.

The Submitting Author accepts and understands that any supply made under these terms is made by BMJ to the Submitting Author unless you are acting as an employee on behalf of your employer or a postgraduate student of an affiliated institution which is paying any applicable article publishing charge ("APC") for Open Access articles. Where the Submitting Author wishes to make the Work available on an Open Access basis (and intends to pay the relevant APC), the terms of reuse of such Open Access shall be governed by a Creative Commons licence – details of these licences and which Creative Commons licence will apply to this Work are set out in our licence referred to above.

Other than as permitted in any relevant BMJ Author's Self Archiving Policies, I confirm this Work has not been accepted for publication elsewhere, is not being considered for publication elsewhere and does not duplicate material already published. I confirm all authors consent to publication of this Work and authorise the granting of this licence.

For peer review only

Associations between autistic traits and early ear and upper respiratory signs: a prospective observational study of the ALSPAC geographically defined childhood population

Amanda Hall[1] (0000-0001-8520-6005), **Richard Maw**[2], **Yasmin Iles-Caven**[3](0000-0002-9965-9133), **Steven Gregory**[3](0000-0002-1529-9338), **Dheeraj Rai**[4] (0000-0002-7239-3523) **and Jean Golding**[3] (0000-0003-2826-3307)

[1]College of Health and Life Sciences, Aston University, Birmingham, UK

[2]University Hospitals Bristol NHS Foundation Trust, Bristol, UK

[3]Centre for Academic Child Health, Population Health Sciences, Bristol Medical School, University of Bristol, Bristol UK

[4]Centre for Academic Mental Health, Bristol Medical School, University of Bristol, Bristol, UK

Correspondence to:

Jean Golding
Centre for Academic Child Health, Bristol Medical School (Population Health Sciences), University of Bristol, Oakfield House, Oakfield Grove, Bristol BS8 2BN, UK
jean.golding@bristol.ac.uk

For peer review only

Abstract

Objective: To determine whether early ear and upper respiratory signs are associated with the development of high levels of autistic traits or diagnosed autism.

Design: Longitudinal birth cohort: Avon Longitudinal Study of Parents and Children (ALSPAC).

Setting: Area centred on the city of Bristol in Southwest England. Eligible pregnant women resident in the area with expected date of delivery between April 1991 and December 1992 inclusive.

Participants: 10,000+ young children followed throughout their first 4 years. Their mothers completed three questionnaires between 18-42 months recording the frequency of nine different signs and symptoms relating to the upper respiratory system, as well as ear and hearing problems.

Outcome measures: Primary – high levels of autism traits (social communication, coherent speech, sociability, and repetitive behaviour); secondary – diagnosed autism.

Results: Early evidence of mouth breathing, snoring, pulling/ poking ears, ears going red, hearing worse during a cold, and rarely listening were associated with high scores on each autism trait and with a diagnosis of autism. There was also evidence of associations of pus or sticky mucus discharge from ears especially with autism and with poor coherent speech. Adjustment for ten environmental characteristics made little difference to the results, and substantially more adjusted associations were at P<0.001 than expected by chance (41 observed; 0.01 expected). For example, for discharge of pus or sticky mucus from ears the adjusted odds ratio (AOR) for autism at 30 months was 3.29 [95%CI:1.85,5.86; P<0.001], and for impaired hearing during a cold AOR=2.18 [1.43,3.31; P<0.001].

Conclusions: Very young children exhibiting common ear and upper respiratory signs appear to have an increased risk of a subsequent diagnosis of autism or demonstrated high levels of autism traits. Results suggest the need for identification and management of ENT conditions in autistic children and may provide possible indicators of causal mechanisms.

Keywords: ALSPAC; autism; early childhood; upper respiratory symptoms; hearing; snoring

For peer review only

Strengths and limitations of this study

- The strength of these results lies in the fact that the data were collected prospectively using a geographically defined population of young children.
- Two of the autism traits were identified using well validated scales (social communication and speech coherence).
- There was no bias in the way in which the questions were asked nor in the later identification of autism or of autism traits.
- A limitation is that not all children in the study were assessed for a diagnosis of autism.

Introduction

Autism spectrum disorder, henceforward named as ‘autism’ is a fairly uncommon, yet heterogeneous, condition.[1] Its aetiology is complex and likely to involve a combination of gene-environment-biology interactions, with no one single cause.[2] There is increasing recognition that the aetiology of each autistic trait may differ, either genetically, epigenetically and/or conditionally upon an environmental exposure.[2]

Understanding and addressing early comorbidities of children diagnosed with autism may lead to longer term improvements in quality of life and also inform the common mechanisms and pathways in the origins of autism.[3] Ear, nose and throat (ENT) including related hearing disorders have been implicated in the development of autism[4 5] including acute otitis media (ear infections), otitis media with effusion (OME or glue ear), temporary conductive hearing loss associated with otitis media, and sleep disordered breathing. For example, there have been a number of studies using population health records, comparing the frequency of ear infections in children with autism compared to controls. Most showed greater odds of ear infections in children with autism: for example, a US population sample of 483 autism children with 84,789 controls found an odds ratio (OR) of 3.1 (95% CI 2.1 to 4.7) for at least three ear infections;[6] others have also shown excesses of ear infections.[7 8] Conversely two studies using Kaiser Permanente health records[9] showed no excess of ear infections among children with autism, but one did find an increased odds (OR 1.93, 95 CI 1.56 to 2.38) of ENT conditions associated with autism compared to controls.[5] Other conditions that have been implicated include sleep-disordered breathing or obstructive sleep apnoea in the early life history of children with autism in the USA[5 10] and Japan.[11] Others have shown an increased likelihood of surgery for a sleep disorder, such as adenoidotonsillectomy.[12]

Of other ENT conditions, there are fewer studies examining early history of chronic OME which is more likely than acute otitis media to interfere with hearing, although less easy to identify from health records. One method is to examine the rate of insertion of ventilation tubes, although it is not always possible to distinguish ventilation tubes inserted for OME from insertion for acute ear infections. Nevertheless, ventilation tube rates have been shown to be higher in children with autism.[7 13 14] Case control studies comparing auditory function in children with autism have found more abnormal tympanograms in

For peer review only

children suggesting a higher prevalence of OME,[15] and more auditory abnormalities.[16] Although other studies did not find more auditory abnormalities in children with autism[17 18]; in both these studies children were excluded if they had a history of ear infections and middle ear effusion.

Overall, therefore, the evidence suggests an increased prevalence of ENT and related hearing conditions in children with autism compared with typically developing children. Much of the evidence cited above is from health records which may be biased due to greater levels of help-seeking from health services of parents of children with suspected autism. Where a parent has concerns about their child's general development, they may be more likely to attend health services, resulting in increased recording of the presence of common health conditions even where the underlying prevalence is no more common than in the general population.

Longitudinal population cohort studies avoid many of the biases in studies using health records, but there are few such studies in the literature apart from that of Jeans and colleagues[19] who analysed data from the Early Childhood Longitudinal Study Birth cohort and found an increased OR of 1.46 [95% CI 1.24, 1.72] of early ear infections in children with a later autism diagnosis. The present study, positioned within the longitudinal birth cohort ALSPAC (Avon Longitudinal Study of Parents and Children), uses information collected prospectively from birth to assess whether any early indications of ENT symptoms, including ear infections and intermittent hearing problems, are associated with one or more of the traits shown to be strongly related to a diagnosis of autism. Its aim is to investigate whether children with autism, and those with high levels of autistic traits or a diagnosis of autism were recorded as having more early ear and upper respiratory signs and symptoms than expected by chance.

Methods

Study population

ALSPAC is a pre-birth cohort study that enrolled ~80% of pregnant women resident in the Avon area of the UK in 1991-2. The initial number of pregnancies enrolled was 14,541 (for these at least one questionnaire had been returned or a "Children in Focus" clinic had been attended by 19/07/99). Of these initial pregnancies, there was a total of 14,676 fetuses, resulting in 14,062 live births and 13,988 children who were alive at 1 year of age. The aim of the study is to assess ways in which the environment (defined in its broadest sense) interacts with genetics to influence the health, development and well-being of the offspring.[20] To this end, data collection used a variety of methodologies including direct examination of the offspring, self-completion questionnaires administered to the parents, the children and their teachers, collection and assays of biological samples (including DNA), linkage to health and education records.[21 22] The study website contains details of all the data that is available through a fully searchable data dictionary and variable search tool: http://www.bristol.ac.uk/alspac/researchers/our-data/. ALSPAC has had its own ethics committee since its inception.[23]

Outcomes

Identification of autism

For peer review only

In order to identify the children with autism we used the following sources: (a) a review of all children given a statement for special educational provision in the Avon area, to identify children diagnosed by age 11 as having special educational needs conforming to a diagnosis of autism using the ICD-10 criteria[24]; (b) the mother's answer to the question at age 9 "Have you ever been told that your child has autism, Asperger's syndrome or autistic spectrum disorder"; (c) classification as Pervasive Development Disorder using questions from the DAWBA questionnaire at 91 months,[25] with the answers to the questionnaire classified by a child psychiatrist; (d) text responses to any question on diagnoses given to the child in questionnaires from 6 months to 11 years suggesting the child had been given an autism diagnosis; (e) *ad hoc* letters from parents to the Study Director. We considered that no one of these sources would be adequate, and so used all, and monitored the overlaps. In this way we identified 177 ALSPAC offspring with a probable diagnosis of autism – 139 boys and 38 girls. Validation of the use of this group as autism was shown by its strong relationship with a polygenic risk score for autism.[26]

<u>Autistic traits</u>

We have used the upper 10th centile of the scores on the four independent trait predictors of autism identified previously as most predictive of autism in this cohort and described below. They included measures of social communication, coherent speech, sociability temperament and repetitive behaviour. Each had been shown to be an independent predictor of autism as identified using clinical records in this cohort.[27]

<u>*Social communication trait*</u>

We used the 12-item Social and Communication Disorders Checklist (SCDC), developed by Skuse and colleagues.[28] They showed that the internal consistency was excellent (0.93) and the test-retest reliability was high (0.81). The method was developed on clinical samples, and when later used on the ALSPAC population at age 7.7 years the high end of the scale was shown to predict a variety of adverse outcomes, but was most specific for autism spectrum disorder.[29] Further research with ALSPAC data showed that the measure was reasonably stable over time.[30] For the present analysis we have used the prorated score, which was calculated when any items of the scale were missing a response, by using the average of the items that had been answered by the individual (2.7% of the population, almost all of whom had just one item missing). If all items were missing, the score was put to missing. The measure ranged from 0 to 24, the higher the score the more impaired was the child's social cognition. The distribution was skewed with a long upper tail (12.8% had a score of over 6 and comprise the group with highest autistic trait for these analyses).

<u>*Coherence measure*</u>

At age 9 the study mother completed a questionnaire which included 7 of the 9 scales of the first version of the Children's Communication Checklist (CCC).[31] This checklist was designed to assess aspects of communication that were not readily identified by conventional standardised tests, including aspects of speech and syntax as well as pragmatic aspects such as over-literal interpretation of stereotyped language. Although the CCC was initially designed to identify pragmatic difficulties, it has been shown to be good at discriminating a wide range of language and communication problems from typical development.[32] Analyses of traits predictive of autism in ALSPAC showed that the Coherence scale performed better than the other CCC scales[27] and consequently it is used here. The scale comprises 8 items (e.g., "It is sometimes hard to make sense of what she is saying because it seems illogical or

For peer review only

disconnected" and "She has difficulty in telling a story or describing what she has done in a sequence of events"). The score ranged from 20 to 36, with higher scores indicating more typical behaviour. The score had a skewed distribution. The lower tail used in this analysis comprised those children scoring <33 points (10.0% of the population).

Abnormal and repetitive behaviour

This scale was developed from the answer to four questions in the questionnaire sent to the mother at 69 months; these were as follows: "How often does he/she: (a) repeatedly rock his head or body for no reason; (b) have a tic or twitch; (c) have other unusual behaviour"; or (d) "Does he/she stumble or get stuck on words, or repeat them many times? (e.g. I I I I want a sweet)"? The responses to each question were coded as: often/always = 3; sometimes = 2; never = 1 and summed. The resultant scale had a range from 4 to 12, with 22% scoring 5 and only 5.9% scoring more than 5. As it was impossible to approximate to a 10% cut-off, we used > 5 as our group with highest autistic trait.

Sociability temperament

The questionnaire concerning the child which was sent to the study mothers when the child was 38 months of age included the 20 questions of the EAS Temperament scale[33]; this measured four traits – emotionality, activity, shyness, and sociability – each based on the answers to 5 questions. The range of the Sociability sub-score was from 5 to 25 and the frequency distribution was approximately normal, a high score indicating a high level of sociability. The prorated scale was calculated for missing values as in the scales mentioned above. We then selected the lowest 11.4% of the children for our analyses (score <8) as being the nearest to 10% as the group with highest autistic trait.

Maternal reports of ear and upper respiratory signs and symptoms

Questions regarding their child's ear and upper respiratory signs were sent to the mother by post when the child was aged: 18, 30 and 42 months. Each questionnaire was structured with response boxes to be ticked. For the current study we used nine questions which were repeated at these three time points, and which described common ENT signs relating to hearing and the upper respiratory system. Details of the actual wording of the questions are shown in Table 1. They cover aspects of breathing (mouth breathing, snoring, symptoms of sleep apnoea), ears (pulling or poking at ears, ears going red, pus discharging from ears), and subjective signs of hearing loss and listening difficulties (hearing worse during a cold, child rarely listens). Questions concerning whether or not the child had had earache were also asked, and the results are provided, but since the children were mostly too young to describe what was wrong, we have treated these data as less reliable than the other questions asked.

For peer review only

Table 1. ENT and hearing related questions in the ALSPAC parental questionnaires

Has anyone thought there may be a problem with his hearing?
Has your baby had earache?
Does s/he breathe through her mouth rather than through her nose?
Does s/he snore for more than a few minutes at a time?
When she is asleep, does s/he seem to stop breathing or hold her breath for several seconds at a time?*
Does she pull, scratch or poke at her ears?
Do his/her ears go red and look sore for a long time?
Has pus or a sticky mucus (not ear wax) ever leaked out of his/her ear?
During or after a cold, is his/her hearing worse than usual?
Generally, does your toddler listen to people or to things that happen nearby?

*Indicating sleep apnoea,

Potential confounders

Adjustments were made for the following ten potential confounders: preterm gestation (<37 weeks; 37+ weeks), sex, parity (defined as the number of previous pregnancies resulting in a live or stillbirth, 0 v 1+), breast feeding (any v none), maternal depression at 8 weeks post-delivery as assessed using the Edinburgh Postnatal Depression Score,[34] maternal educational achievements (5-point scale), maternal smoking at 18 weeks gestation (none v any), maternal locus of control (using a shortened version of the adult version of the Nowicki-Strickland Internal-External locus of control scale as described elsewhere,[35] child's exposure to environmental tobacco smoke (ETS) at 15 months measured as the length of time the child was in a room with others smoking at weekends and weekdays (any v none) and attending a crèche or other type of day care by 30 months (yes v no). Table 2 shows frequencies of these confounders.

Table 2. Frequencies of variables used as confounders

Confounder	No. with data available	Frequency
Pre-term delivery	12,935	5.3% <37 weeks
Sex of child	14,676	51.8% male
Parity of mother	12,787	48.2% Primiparous
Whether breast fed at 7 days	11,880	66.1%
Maternal depression at 2m	11,213	8.8%
Maternal education level (5 levels)	12,370	From 20.8% lowest to 12.8% highest
Maternal smoking at 18 weeks gestation	13,274	19.6%
Maternal locus of control	12,471	45.8% external
Child's exposure to environmental tobacco smoke at 15m	11,073	42.0% exposed
Attended creche or equivalent by 3y	10,038	36.3% exposed

Statistical analysis

For peer review only

Initial analysis used unadjusted comparisons between the different signs and symptoms and the five different binary outcomes (the most disadvantaged 10% of the four autistic traits and diagnosed autism). Logistic regression using STATA was then used to allow for the 10 potential confounders – with 120 logistic regressions being undertaken. In order to take account of multiple testing we determined how many associations would be expected to result in P<0.01 by chance for both the unadjusted and the adjusted associations. For each set of analyses just 1.2 of the 120 results were expected at P<0.01. Any excess in numbers of adjusted results at P<0.001 were assumed to be either indicating causality or the possibility of unadjusted confounding.

Patient and public involvement

Patients and/or the public were not involved in the reporting or dissemination plans of this research. Prior to data collection (in the early 1990s), focus groups of pregnant women were involved in discussions on the content of questionnaires.

Results

Unadjusted comparisons of ear and upper respiratory signs with autism outcomes

The responses to each question asked at 18, 30 and 42 months are compared between those scoring at the extreme ends of the autism trait scores, as well as for those diagnosed with autism in Supplementary Tables 1-8. Of the 120 comparisons, 85 were at P<0.01 (1.2 expected) and 66 at P<0.0001 (0.001 expected).

Among the different ages tested, the most striking were at age 30 months (Table 3) where it can be seen that those groups with the highest autistic traits reported more ear and upper respiratory signs. It is noteworthy that autism itself was significantly associated with all signs except for symptoms of sleep apnoea. Conversely, the sociability trait was much less strongly associated with these ear and upper respiratory signs than any of the other three traits. Also note that pus leaking from the ears was only associated with abnormal scores on the speech coherence trait and with autism itself.

Table 3. Summary of unadjusted results comparing % rates (n) of ENT signs at 30 months of children who later demonstrated high levels of autistic traits and of children diagnosed with autism contrasted with controls

Signs and symptoms	Group	Social communication % (n)	Speech Coherence % (n)	Sociability %(n)	Repetitive behaviour % (n)	Autism % (n)
Mouth breathing	Index	27.2 (232)	26.5 (169)	24.5 (226)	24.1 (489)	30.3 (42)
	Control	19.1 (1119)	19.8 (1145)	20.8 (1524)	19.0 (992)	21.8 (1966)
	P value	***	***	*	***	**
Snoring	Index	25.4 (221)	22.0 (150)	20.9 (196)	20.2 (417)	31.0 (41)
	Control	16.0 (947)	16.6 (967)	17.4 (1283)	16.1(851)	18.2 (1660)
	P value	***	***	**	***	***
Sleep apnoea	Index	18.1 (148)	18.4 (110)	15.6 (143)	17.4 (339)	16.8 (21)
	Control	14.0 (802)	13.9 (789)	15.2 (1099)	13.6 (705)	15.5 (1387)

	P value	***	***		***	
Pulling at ears	Index	8.2 (78)	8.4 (60)	6.8 (71)	7.0 (157)	16.2 (25)
	Control	4.1 (267)	4.2 (268)	4.4 (353)	3.7 (213)	4.6 (463)
	P value	***	***	**	***	***
Ears red and sore	Index	21.0 (221)	20.4 (139)	18.0 (181)	19.2 (418)	26.5 (40)
	Control	13.0 (820)	13.5 (841)	14.6 (1150)	12.1(680)	15.0 (1458)
	P value	***	***	**	***	***
Pus/mucus from ears	Index	3.9 (37)	5.1 (36)	4.4 (46)	4.0 (90)	10.3 (16)
	Control	3.2 (207)	3.0 (189)	3.3 (263)	3.1(177)	6.6 (349)
	P value		**			***
Hearing worse during cold	Index	21.5 (148)	25.8 (133)	16.5 (127)	20.1 (319)	37.9 (44)
	Control	14.1 (682)	14.1 (673)	15.6 (946)	13.1(574)	15.2 (1136)
	P value	***	***		***	***
Rarely listens	Index	4.6 (44)	6.4 (45)	5.1 (53)	4.1 (91)	14.4 (22)
	Control	1.6 (101)	1.5 (95)	1.7 (139)	1.4 (80)	2.1 (213)
	P value	***	***	***	***	***

*P< 0.05; ** P < 0.01; *** P < 0.001.

Adjusted comparisons of ear and upper respiratory signs with autism outcomes

Results of adjustment for 10 factors (gestation, sex, parity, breast feeding, maternal depression, education, prenatal smoking, maternal locus of control, child exposure to environmental tobacco smoke, and starting crèche by age 30m) are shown in Table 4. It can be seen that of the 120 logistic regression results, 41 were associated at P<0.001 (0.0012 expected). The results are discussed in terms of the three categories of ear and upper respiratory signs below.

Table 4. High levels of autistic traits or of diagnosed autism by upper respiratory signs
Odds ratios [95% confidence intervals] adjusted for gestation, sex, parity, breast feeding, maternal depression, maternal education, prenatal smoking, locus of control, child exposure to environmental tobacco smoke and starting crèche (see main text for details).

**Outcome**	**Social communication AOR [95% CI]**	**Speech Coherence AOR [95% CI]**	**Sociability AOR [95% CI]**	**Repetitive behaviour AOR [95% CI]**	**Autism AOR [95% CI]**
Mouth breathing					
18m	1.12 [0.90,1.39]	1.51 [1.21,1.81]***	1.34 [1.07,1.67]**	1.36 [1.14,1.62]***	2.13 [1.32,3.44]**
30m	1.41 [1.20,1.67]***	1.31 [1.10,1.55]**	1.11 [0.93,1.33]	1.24 [1.09,1.43]**	1.62 [1.07,2.43]*
42m	1.43 [1.22,1.67]***	1.32 [1.12,1.57]***	1.09 [0.92,1.30]	1.31 [1.15,1.49]***	1.40 [0.93,2.11]
Snoring					

For peer review only

18m	1.72 [1.23,2.41]**	1.92 [1.35,2.72]***	1.12 [0.76,1.64]	1.31 [0.97,1.77]	1.38 [0.55,3.43]
30m	1.54 [1.18,2.03]**	1.32 [0.98,1.78]	1.13 [0.83,1.53]	1.21 [0.95,1.52]	1.54 [0.77,3.11]
42m	1.47 [1.15,1.88]**	1.33 [1.02,1.73]*	1.00 [0.75,1.33]	1.20 [0.97,1.48]	1.93 [1.09,3.41]*
Sleep apnoea					
18m	1.54 [1.20,1.96]***	1.23 [0.93,1.62]	0.89 [0.66,1.19]	1.33 [1.08,1.63]**	1.14 [0.57,2.28]
30m	1.24 [1.02,1.50]*	1.30 [1.06,1.59]*	0.91 [0.73,1.13]	1.17 [1.00,1.37]*	0.93 [0.53,1.63]
42m	1.28 [1.06,1.55]**	1.33 [1.09,1.62]**	0.99 [0.80,1.23]	1.20 [1.03,1.41]*	1.35 [0.84,2.17]
Pulls/pokes at ears					
18m	1.42 [1.13,1.79]**	1.11 [0.86,1.44]	1.24 [0.96,1.59]	1.42 [1.17,1.73]***	1.34 [0.75,2.41]
30m	1.57 [1.20,2.07]***	1.52 [1.14,2.03]**	1.48 [1.10, 1.99]**	1.78 [1.41,2.25]***	3.40 [2.06,5.64]***
42m	1.33 [0.93,1.90]	1.92 [1.38,2.69]***	1.74 [1.24,2.46]***	1.52 [1.14,2.03]**	3.77 [2.11,6.74]***
Ears turn red					
18m	1.19 [1.01,1.40]*	1.09 [0.92,1.29]	1.13 [0.95,1.34]	1.46 [1.28,1.66]***	1.10 [0.72,1.69]
30m	1.35 [1.13,1.61]***	1.17 [0.96,1.41]	1.20 [0.99,1.45]	1.54 [1.33,1.79]***	2.14 [1.44,3.17]***
42m	1.27 [1.05,1.53]*	1.21 [0.99,1.47]	1.05 [0.86,1.30]	1.33 [1.14,1.56]***	1.52 [0.98,2.37]
Pus/mucus from ears					
18m	0.98 [0.65,1.48]	1.27 [0.86,1.90]	1.26 [0.86,1.85]	1.14 [0.83,1.57]	1.87 [0.85,4.08]
30m	1.12 [0.78,1.60]	1.32 [0.92,1.89]	1.19 [0.83,1.71]	1.33 [1.00,1.75]*	3.29 [1.85,5.86]***
42m	1.31 [1.01,1.72]*	1.23 [0.93,1.64]	1.22 [0.91,1.65]	1.31 [1.05,1.64]*	2.85 [1.72,4.72]***
Hearing worse during a cold					
18m	1.22 [0.88,1.71]	1.53 [1.10,2.14]*	1.22 [0.86,1.73]	1.25 [0.95,1.64]	0.82 [0.33,2.06]
30m	1.33 [1.10,1.62]**	1.35 [1.11,1.65]**	1.22 [1.00,1.49]	1.40 [1.20,1.64]***	2.18 [1.43,3.31]***
42m	1.47 [1.20,1.79]***	1.73 [1.41,2.12]***	1.10 [0.88,1.37]	1.59 [1.35,1.88]***	3.28 [2.15,5.00]***
Rarely listens to nearby sounds					
18m	2.40 [1.61,3.58]***	1.58 [1.00,2.51]	2.07 [1.40,3.06]***	1.89 [1.32,2.72]***	2.26 [0.97,5.27]
30m	2.56 [1.76,3.73]***	2.69 [1.82,3.97]***	2.87 [2.01,4.10]***	2.57 [1.84,3.59]***	6.44 [3.68,11.28]***
42m	2.64 [1.96,3.57]***	2.76 [2.01,3.79]***	2.31 [1.70,3.15]***	2.11 [1.60,2.79]***	6.60 [4.04,10.77]***

*P< 0.05; ** P < 0.01; *** P < 0.001, AOR = adjusted odds ratio.

Differences in breathing

For peer review only

The unadjusted comparisons showed that mouth breathing (all of the time or much of the time) was positively associated with autistic traits and autism at each of the three ages at which the question was asked, with P<0.05 for 14 of the 15 data points. Similarly, snoring at each of the three time points showed positive associations with the autism outcomes, all of the 15 comparisons being at P<0.05. However, for symptoms of sleep apnoea (often or sometimes), although there were always more affected children with the autistic outcome, only 10 of 15 associations showed differences at P<0.05 (Supplementary Tables 1-3).

On adjustment (Table 4) the three signs in this category showed different associations: social communication and coherence showed associations with all three signs, repetitive behaviour with two of the signs (mouth breathing and sleep apnoea) but sociability only with mouth breathing and only at one time point (18 months). Although numbers with autism were small, the adjusted odds ratio (AOR) was as high as 1.93 and 2.13 for mouth breathing and snoring respectively.

Signs of ear differences

The proportion of children who often pulled, scratched, or poked at their ears also showed positive unadjusted associations at P<0.05 in 11 of the 15 autism outcomes, and 14 of 15 associations for a history of the child's ears being "red and sore looking". "Pus leaking from the ear (more than once)" was associated with lower number of autistic outcomes (7 of 15), with particularly strong associations with speech coherence and autism (Supplementary Tables 4-6).

After adjustment (Table 4), pulling, scratching, or poking at ears was particularly strongly associated with autism, with AORs of 3.40 (95%CI 2.06, 5.640 and 3.77 (95%CI 2.11, 6.74) at 30 and 42 months. There were also strong associations for each autistic trait: the age at maximum odds varied from 30 months for social communication and repetitive behaviour, to 42 months for speech coherence and sociability. The adjusted odds were less marked for children whose ears were reported to have gone red for prolonged periods of time and were only associated with two traits (social communication and repetitive behaviour). For autism the association was strongest at 30m with an AOR of 2.14 (95% CI 1.44, 3.17). For pus from the ears, there were only mild associations with the autistic traits, but strong associations with autism, particularly at 30 months with an AOR of 3.29. (95%CI 1.85, 5.86).

Signs of listening and hearing difficulties

On unadjusted analyses, reports of the child's hearing being worse during or after a cold were associated with autism and for three of the four autistic traits (sociability being the exception). The differences of the 4 outcomes were apparent at each of the three ages at P<0.0001 (Supplementary Table 7). The unadjusted pattern was different in relation to the question concerning whether the child listens to people or to things that happen nearby. Here all comparisons were at P<0.05, including the poor sociability outcome (Supplementary Tables 7 and 8).

On adjustment, the results are similar to the unadjusted associations (Table 4), with the AORs being particularly high for failure to react to noise nearby at 30 months of age for children with autism [AOR = 6.44, 95% CI 3.68, 11.28; P<0.0001].

Discussion

For peer review only

There is increasing interest worldwide in autism, and much research has been undertaken to understand the neuro-cognitive and genetic differences.[36] In this set of analyses we have shown that common indicators of ear health and upper respiratory compromise are more frequent in children subsequently identified with autism as well as in children who have high levels of several autistic traits. These associations may be important because (a) these ear and respiratory signs may be early markers of increased risk of autism; (b) they may inform the origins of autism; or (c) that they may highlight co-occurring conditions that if treated may lead to a better quality of life for children with autism.

No birth cohort studies, to our knowledge, have investigated prospectively the common signs that might indicate upper respiratory disadvantage such as snoring, mouth breathing or sleep apnoea as precursors of autism. Sleep problems are commonly reported by parents of children with autism, including problems due to sleep-related breathing disorders, such as snoring or apnoea.[37] In this study we found that mouth breathing in the first three years of life was associated with autism, but not symptoms of sleep apnoea, or early snoring except at 42 months. However, both snoring and symptoms of sleep apnoea in the early years were linked to the autistic traits of social communication and speech coherence, across most of the time points; sleep apnoea was also linked to repetitive behaviour. Studies examining whether sleep related breathing disorders are more commonly reported in autism than in typically developing children are generally case-control studies of children with a confirmed diagnosis of autism. Results generally show more sleep disordered breathing in children with autism,[10 11 38 39 40] although Alfonso-Alfonso et al.[41] found no difference and Malow and colleagues[42] carried out polysomnography in children with autism and typically developing controls and found no physiological evidence of sleep apnoea in the autism group. Our findings based on a large sample size may indicate that children who develop autistic traits have a slightly disordered upper respiratory structure or physiology, such as obstructed airways from enlarged adenoids. For example, snoring results during sleep when the upper respiratory system relaxes and various organs in the airways vibrate and breathing through the mouth rather than the nose is an indicator of obstructed airways, as is breath holding or stopping breathing during sleep.

Similarly, subtle signs of possible middle ear disease such as pulling or poking at the ears, the ears going red for a prolonged period of time, or pus or sticky mucus discharging from the ears appear not to have been considered before in birth cohort studies. We found that these signs were closely associated with autistic traits and with a diagnosis of autism, especially if the signs were present at around 30 months of age. There were also strong relationships between ear discharge and autism. Ear infections have been linked to autism based on medical attendance for otitis media in health registry studies,[4 7 8] through prospective parental report of ear infections in a birth cohort study[19] and retrospective parental report in a case-control study.[14] Interestingly, a study based on the Danish National Birth Cohort found no association between parental report of ear infections and autism, but did find an association when hospital contact for ear infections was examined as the exposure[43]; the authors suggest the hospital record data could be affected by both detection and selection bias which is less likely to influence studies involving prospective parental reporting. In order to determine whether the child's hearing was affected in the absence of direct hearing tests, we assessed the responses to questions concerning whether the child's hearing was thought by the mother to be worse during a cold, and whether she reported that the child rarely listened to people or things that happened nearby. Both characteristics were associated with autism and the autistic traits, particularly at 30 months. Although unresponsive behaviour is a feature of the social communication autistic trait, the associations

For peer review only - http://bmjopen.bmj.com/site/about/guidelines.xhtml

with listening difficulties accompanying a cold implies the presence of a fluctuating conductive hearing loss rather than inattention. This finding is consistent with case-control studies that have shown higher levels of abnormal tympanograms (middle ear function tests indicating the presence of otitis media) in autism.[15 44 45] There is limited evidence however that older children with autism have peripheral hearing loss, with relevant studies systematically reviewed in 2014.[46]

This study adds to the evidence that, compared to a typical population of the same age, early ear and upper respiratory symptoms are more common in those subsequently diagnosed with autism or with extreme levels of autistic traits. It is not possible to determine whether these ENT conditions have a causal role in the development of autistic traits or are related to an unmeasured factor. One possibility, for example, could be the consequence of the increased prevalence of minor physical anomalies in individuals with autism,[47] including anatomical differences in the structure and/or positioning of the ear[14 48] with such differences in ear morphology increasing the risk of ENT conditions.

Ongoing otitis media with effusion during early childhood leads to impairment in auditory processing, although this improves as otitis media clears.[49] Atypical sensory processing is a common presentation in children with autism,[50 51] but also the prevalence of autism is higher in deaf children than hearing children and similarly higher in children with visual impairment.[52] This suggests that in children with an existing susceptibility to development of autism, disrupted auditory processing as a result of otitis media may interact with existing deficiencies in sensory processing and integration, impacting on development of autistic traits.[50]

Aside from suggestions as to mechanisms, it is clear from this study of prospectively collected information, that children who later develop social communication difficulties are more likely to have early middle ear disease and ENT conditions and are therefore more at risk of communication difficulties from hearing loss, albeit temporary. Early detection and intervention of ENT conditions in children with autism is thus likely to be beneficial.

Strengths and limitations

An important strength of the study lies in the fact that the population is geographically defined and includes the great majority (~80%) of the eligible population. The data are possibly unique in documenting common early ear and upper respiratory signs and symptoms of the children who subsequently develop extreme levels of autistic traits and/or a diagnosis of autism. The mothers answered structured questions at distinct time points with no idea as to whether the signs they were describing were linked to outcomes such as autism. Thus, there were no discernible biases in ascertainment of these signs, or of the later identification of the autistic traits.

The limitations, as in all longitudinal studies, lie in the loss of children to later follow up.[21] Unfortunately, at the time the children were born there were very few families of ethnic minorities resident in the area (~6%). Consequently, our results cannot be extrapolated to cover non-white populations in general. Another limitation concerns the fact that, to our knowledge, the questions used to record ear and upper respiratory symptoms have not been validated. However, they ask about objective rather than subjective signs and symptoms, and parents gave similar results over the childhood years. A further limitation concerns the fact that the study children were not examined consistently to determine a diagnosis of autism. Rather a strategy to assess the probability of a diagnosis using a variety of different sources

For peer review only

was used. The validity of this approach was shown since the group of children identified in this way demonstrates a positive correlation with a polygenic risk score for autism.

It is always possible that the statistical analyses did not allow for all the appropriate confounders, and further data sets are needed to assess the validity of our results.

Conclusion

Data collected longitudinally from 18 to 42 months has demonstrated close associations of common early ear and upper respiratory markers with a later diagnosis of autism as well as with high levels of autistic traits. These markers included snoring and mouth breathing as well as a reduction in hearing ability during a cold, and signs of otitis media with infusion. The data indicate that the strongest associations occurred at age 30 months. These results may indicate disruptions in early auditory perception and processing in the early stages of autism.

*** ***

Data availability statement

ALSPAC data is available to researchers for particular projects, provided no attempt is made to reveal the identities of the subjects. Guidelines for access are found on the ALSPAC website (www.bristol.ac.uk/alspac/researchers).

Ethics

Ethical approval for the study was obtained from the ALSPAC Ethics and Law Committee (ALEC; IRB00003312) and the Local Research Ethics Committees. Detailed information on the ways in which confidentiality of the cohort is maintained may be found on the study website: http://www.bristol.ac.uk/alspac/researchers/research-ethics/

All methods were performed in accordance with the relevant guidelines and regulations. Informed consent for the use of data collected via questionnaires and clinics was obtained from participants following the recommendations of the ALSPAC Ethics and Law Committee at the time.[23]

Acknowledgements

We are extremely grateful to all the families who took part in this study, the midwives for their help in recruiting them, and the whole ALSPAC team, which includes interviewers, computer and laboratory technicians, clerical workers, research scientists, volunteers, managers, receptionists, and nurses.

Contributors

Conception and design of study: AH and JG. Acquisition of funding: JG and YIC. Preparation of study data and analyses: AH and SG. Statistical analysis quality checking: JG and AH. Original draft: AH and JG. JG, AH, RM, DR, and YIC contributed to interpretation of the findings. JG, AH, RM, DR, YIC, and SG contributed to the critical review and editing of the manuscript and approved the final manuscript. All authors are guarantors for this work. The corresponding author attests that all listed authors meet authorship criteria and that no others meeting the criteria have been omitted.

Funding

This publication was made possible through the support of grants from the Medical Research Council (G0400085) and the John Templeton Foundation (ref no. 61917). The UK Medical Research Council and Wellcome Trust (Grant ref: 217065/Z/19/Z) and the University of Bristol (grant ref: N/A) currently provide core support for ALSPAC. This publication is the work of the authors who will serve as guarantors for the contents of this paper. A comprehensive list of grants funding is available on the ALSPAC website (http://www.bristol.ac.uk/alspac/external/documents/grant-acknowledgements.pdf). The opinions expressed in this publication are those of the author(s) and do not necessarily reflect the views of the John Templeton Foundation.

Competing interests

JG and RM are retired; YIC and SG are funded by *the John Templeton Foundation (*61917). The authors declare no financial relationships with any organisations that might have an interest in the submitted work in the previous three years and no other relationships or activities that could appear to have influenced the submitted work.

Provenance and peer review

Not commissioned; externally peer reviewed.

For peer review only

References

1. Lai MC, Lombardo MV, Baron-Cohen S. Autism. *Lancet* 2014;**383**:896-910.
2. Happe F, Ronald A, Plomin R. Time to give up on a single explanation for autism. *Nat Neurosci,* 2006;**9**:1218-20.
3. Tye C, Runicles AK, Whitehouse AJO, et al. Characterizing the interplay between autism spectrum disorder and comorbid medical conditions: an integrative review. *Front Psychiatry* 2018;**9**:751
4. Doshi-Velez F, Ge Y, Kohane I. Comorbidity clusters in autism spectrum disorders: an electronic health record time-series analysis. *Pediatrics* 2014;**133**:e54-63.
5. Alexeeff SE, Yau V, Qian Y, et al. Medical conditions in the first years of life associated with future diagnosis of asd in children. *J Autism Dev Disord* 2017;**47**:2067-79.
6. Gurney JG, McPheeters ML, Davis MM. Parental report of health conditions and health care use among children with and without autism: national survey of children's health. *Arch Pediatr Adolesc Med* 2006;**160**:825-
7. Adams DJ, Susi A, Erdie-Lalena CR, et al. Otitis media and related complications among children with autism spectrum disorders. *J Autism Dev Disord* 2016; **46**:1636-42.
8. Cawthorpe D. A 16-year cohort analysis of autism spectrum disorder-associated morbidity in a pediatric population. *Front Psychiatry* 2018;**9**:635.
9. Rosen NJ, Yoshida CK, Croen LA. Infection in the first 2 years of life and autism spectrum disorders. *Pediatrics* 2017;**119**:e61-9.
10. Hodge D, Carollo TM, Lewin M, et al. Sleep patterns in children with and without autism spectrum disorders: developmental comparisons. *Res Dev Disabil* 2014;**35**:1631-8.
11. Hirata I, Mohri I, Kato-Nishimura K, et al. Sleep problems are more frequent and associated with problematic behaviors in preschoolers with autism spectrum disorder. *Res Dev Disabil,* 2016;**49-50**:86-99.
12. Elrod MG, Nylund CM, Susi AL, et al. Prevalence of diagnosed sleep disorders and related diagnostic and surgical procedures in children with autism spectrum disorders. *J Dev Behav Pediatr* 2016;**37**: 377-84
13. Ackerman S, Reilly B, Bernier R. Tympanostomy tube placement in children with autism. *J Dev Behav Pediatr* 2012;**33**:252-8.
14. Konstantareas MM, Homatidis S. Ear infections in autistic and normal children. *J Autism Dev Disord* 1987;**17**:585-94.
15. Rafal Z. Conductive hearing loss in children with autism. *Eur J Pediatr* 2013;**172**:1007-10.
16. Demopoulos C, Lewine JD. Audiometric profiles in autism spectrum disorders: does subclinical hearing loss impact communication? *Autism Res* 2016;**9**:107-20.
17. Gravel JS, Dunn M, Lee WW, et al. Peripheral audition of children on the autistic spectrum. *Ear Hear* 2016;**27**:299-312.
18. Tharpe AM, Bess FH, Sladen DP, et al. Auditory characteristics of children with autism. *Ear Hear* 2006;**27**:430-41.
19. Jeans LM, Santos RM, Laxman DJ, et al. Early predictors of asd in young children using a nationally representative data set. *J Early Interven* 2013;**35**:303-31.
20. Golding J, Pembrey M, Jones R. ALSPAC--the avon longitudinal study of parents and children. I. Study methodology. *Paediatr Perinat Epidemiol* 2001;**15**:74-87.
21. Boyd A, Golding J, Macleod J, et al. Cohort profile: the 'children of the 90s'--the index offspring of the avon longitudinal study of parents and children. *Int J Epidemiol* 2013;42:111-27.

For peer review only

22. Fraser A, Macdonald-Wallis C, Tilling K, et al. Cohort Profile: The Avon Longitudinal Study Of Parents And Children: ALSPAC mothers cohort. *Int J Epidemiol* 2013;**42**:97-110.
23. Birmingham K. Pioneering ethics in longitudinal studies: The early development of the ALSPAC Ethics & Law Committee. Policy Press, 2018.
24. Williams E, Thomas K, Sidebotham H, et al. Prevalence and characteristics of autistic spectrum disorders in the ALSPAC cohort. *Dev Med Child Neurol* 2008; **50**:672-7.
25. Goodman R, Ford T, Richards H, et al. The development and well-being assessment: description and initial validation of an integrated assessment of child and adolescent psychopathology. *J Child Psychol Psychiatry* 2000;**41**:645-55.
26. Rai D, Culpin I, Heuvelman H, et al. Association of autistic traits with depression from childhood to age 18 years. *JAMA Psychiatry* 2018;**75**:835-43.
27. Steer CD, Golding J, Bolton PF. Traits contributing to the autistic spectrum. *Plos One* 2010;**5**:e12633.
28. Skuse DH, Mandy WP, Scourfield J. Measuring autistic traits: heritability, reliability and validity of the Social And Communication Disorders Checklist. *Br J Psychiatry,* 2005;**187**:568-72.
29. Skuse DH, Mandy W, Steer C, et al. Social communication competence and functional adaptation in a general population of children: preliminary evidence for sex-by-verbal IQ differential risk. *J Am Acad Child Adolesc Psychiatry* 2009;**48**:128-37.
30. St Pourcain B, Mandy WP, Heron J, et al. Links between co-occurring social-communication and hyperactive-inattentive trait trajectories. *J Am Acad Child Adolesc Psychiatry* 2011;**50**:892-902.e5
31. Bishop DV. Development of the children's communication checklist (ccc): a method for assessing qualitative aspects of communicative impairment in children. *J Child Psychol Psychiatry* 1998;**39**:879-91.
32. Bishop DV, Baird G. Parent and teacher report of pragmatic aspects of communication: use of the children's communication checklist in a clinical setting. *Dev Med Child Neurol* 2001;**43**:809-18.
33. Buss AH, Plomin R. Temperament: early developing personality traits. L Erlbaum Associates, 1984.
34. Cox JL, Holden JM, Sagovsky R. Detection of postnatal depression. Development of the 10-item Edinburgh Postnatal Depression Scale. *Br J Psychiatry* 1987;**150**:782-6.
35. Golding J, Gregory S, Iles-Caven Y, et al. The antecedents of women's external locus of control: Associations with characteristics of their parents and their early childhood. *Heliyon* 2017;**3**:e00236.
36. Lyall K, Croen L, Daniels J, et al. The changing epidemiology of autism spectrum disorders. *Ann Rev Public Health* 2017;**38**:81-102.
37. Schreck KA, Richdale AL. Sleep problems, behavior, and psychopathology in autism: inter-relationships across the lifespan. *Curr Opin Psychol* 2020;**34**:105-11.
38. Schreck KA, Mulick JA. Parental report of sleep problems in children with autism. *J Autism Dev Disord* 2000;**30**:127-35.
39. Trickett J, Heald M, Oliver C, et al. A cross-syndrome cohort comparison of sleep disturbance in children with Smith-Magenis syndrome, Angelman syndrome, autism spectrum disorder and tuberous sclerosis complex. *J Neurodev Disord* 2018;**10**:9.
40. Mutluer T, Karakoc Demirkaya S, Abali O. Assessment of sleep problems and related risk factors observed in Turkish children with Autism spectrum disorders. *Autism Res* 2016;**9**:536-42.

For peer review only

41. Alfonso-Alfonso M, Morales-Chacón LM, González-Naranjo JE. Subjective assessment of sleep in infantile autism: a comparative study. *Behav Sci (Basel)* 2019;**9**
42. Malow BA, Marzec ML, McGrew SG, et al. Characterizing sleep in children with autism spectrum disorders: a multidimensional approach. *Sleep* 2006;**29**:1563-71.
43. Atladottir HO, Henriksen TB, Schendel DE, et al. Using maternally reported data to investigate the association between early childhood infection and autism spectrum disorder: the importance of data source. *Paediatr Perinat Epidemiol* 2012;**26**:373-85.
44. Rosenhall U, Nordin V, Sandstrom M, et al. Autism and hearing loss. *J Autism Dev Disord* 1999;**29**:349-57.
45. Smith DE, Miller SD, Stewart M, et al. Conductive hearing loss in autistic, learning-disabled, and normal children. *J Autism Dev Disord* 1988;**18**:53-65.
46. Beers AN, McBoyle M, Kakande E, et al. Autism and peripheral hearing loss: a systematic review. *Int J Pediatr Otorhinolaryngol* 2014;**78**: 96-101.
47. Ozgen HM, Hop JW, Hox JJ, et al. Minor physical anomalies in autism: a meta-analysis. *Mol Psychiatry* 2010;**15**:300-7.
48. Rodier PM, Bryson SE, et al. Minor malformations and physical measurements in autism: data from Nova Scotia. *Teratol* 1997;**55**:319-25.
49. Moore DR, Hartley DE, Hogan SC. Effects of otitis media with effusion (OME) on central auditory function. *Int J Pediatr Otorhinolaryngol* 2003;**67**:S63-7.
50. Thye MD, Bednarz HM, Herringshaw AJ, et al. The impact of atypical sensory processing on social impairments in autism spectrum disorder. *Dev Cogn Neurosci* 2018; **29**:151-67.
51. O'Connor K. Auditory processing in autism spectrum disorder: a review. *Neurosci Biobehav Rev* 2012;**36**:836-54.
52. Do B, Lynch P, Macris EM, et al. Systematic review and meta-analysis of the association of Autism Spectrum Disorder in visually or hearing impaired children. *Ophthalmic Physiol Opt* 2017;**37**:212-24.

For peer review only

Hearing and autism: Supplementary Tables

Supplementary Table 1. Comparison of the prevalence of **mouth breathing** among index children (i.e. with high levels of autistic traits or diagnosed autism) and control children (i.e. all who were not in the index category)

Age		SCDC % (n)	Coherence % (n)	Sociability % (n)	Repetitive % (n)	Autism % (n)
18m	Index	13.1 (117)	15.1 (99)	15.1 (143)	13.7 (289)	17.4 (24)
	Control	10.2 (627)	10.5 (633)	11.4 (875)	9.9 (540)	12.2 (1196)
	P	**	****	****	****	*
30m	Index	27.2 (232)	26.5 (169)	24.5 (226)	24.1 (489)	30.3 (42)
	Control	19.1 (1119)	19.8 (1145)	20.8 (1524)	19.0 (992)	21.8 (1966)
	P	****	****	*	****	**
42m	Index	26.9 (260)	25.4 (185)	23.3 (250)	24.9 (573)	25.5 (38)
	Control	18.6 (1219)	18.9 (1228)	20.3 (1690)	18.8 (1013)	20.7 (2054)
	P	****	****	*	****	-

*P< 0.05; ** P < 0.01; *** P < 0.001; **** P < 0.0001

Supplementary Table 2. Comparison of the prevalence of **snoring** among index children (i.e. with high levels of autistic traits or diagnosed autism) and control children (i.e. all who were not in the index category)

Age		SCDC % (n)	Coherence % (n)	Sociability % (n)	Repetitive % (n)	Autism % (n)
18m	Index	15.3 (144)	14.6 (102)	11.8 (122)	11.5 (258)	16.3 (24)
	Control	7.6 (495)	7.9 (511)	8.8 (719)	7.3 (427)	9.5 (998)
	P	****	****	**	****	*
30m	Index	25.4 (221)	22.0 (150)	20.9 (196)	20.2 (417)	31.0 (41)
	Control	16.0 (947)	16.6 (967)	17.4 (1283)	16.1(851)	18.2 (1660)
	P	****	****	**	****	***
42m	Index	24.6 (238)	23.2 (169)	22.2 (237)	22.0 (506)	26.2 (39)
	Control	17.6 (1152)	17.9 (1158)	18.5 (1534)	17.3 (1020)	19.1 (1889)
	P	****	****	**	****	*

*P< 0.05; ** P < 0.01; *** P < 0.001; **** P < 0.0001

For peer review only

Supplementary Table 3. Comparison of the prevalence of possible **sleep apnoea** among index children (i.e. with high levels of autistic traits or diagnosed autism) and control children (i.e. all who were not in the index category).

**Age**		**SCDC % (n)**	**Coherence % (n)**	**Sociability % (n)**	**Repetitive % (n)**	**Autism % (n)**
18m	Index	10.8 (91)	8.8 (54)	8.2 (77)	9.3 (189)	9.9 (13)
	Control	6.8 (405)	7.0 (409)	7.9 (590)	6.9 (367)	7.9 (769)
	P	****	*	-	**	-
30m	Index	18.1 (148)	18.4 (110)	15.6 (143)	17.4 (339)	16.8 (21)
	Control	14.0 (802)	13.9 (789)	15.2 (1099)	13.6 (705)	15.5 (1387)
	P	***	***	-	****	-
42m	Index	16.4 (158)	17.4 (126)	13.8 (148)	15.4 (355)	18.8 (28)
	Control	12.2 (799)	12.1 (784)	12.9 (1073)	12.0 (709)	14.2 (1298)
	P	***	****	-	****	*

*P< 0.05; ** P < 0.01; *** P < 0.001; **** P < 0.0001

Supplementary Table 4. Comparison of the prevalence of **often pulling and poking at ears** among index children (i.e. with high levels of autistic traits or diagnosed autism) and control children (i.e. all who were not in the index category).

**Age**		**SCDC % (n)**	**Coherence % (n)**	**Sociability % (n)**	**Repetitive % (n)**	**Autism % (n)**
18m	Index	10.4 (103)	10.6 (77)	9.3 (99)	9.8 (226)	10.9 (17)
	Control	6.9 (460)	6.9 (456)	7.5 (634)	6.6 (395)	7.9 (862)
	P	****	****	*	****	-
30m	Index	8.2 (78)	8.4 (60)	6.8 (71)	7.0 (157)	16.2 (25)
	Control	4.1 (267)	4.2 (268)	4.4 (353)	3.7 (213)	4.6 (463)
	P	****	****	**	****	****
42m	Index	1.8 (17)	2.8 (20)	2.2 (24)	2.5 (58)	10.7 (16)
	Control	1.8 (116)	1.8 (115)	2.0 (163)	1.8 (106)	5.3 (521)
	P	-	-	-	*	***

*P< 0.05; ** P < 0.01; *** P < 0.001; **** P < 0.0001

For peer review only

Supplementary Table 5. Comparison of the prevalence of **ears going red and sore looking for a long time** among index children (i.e. with high levels of autistic traits or diagnosed autism) and control children (i.e. all who were not in the index category).

**Age**		**SCDC**	**Coherence**	**Sociability**	**Repetitive**	**Autism**
18m	Index	27.3 (261)	27.4 (194)	24.9 (259)	26.6 (602)	25.3 (38)
	Control	19.6 (1290)	19.9 (1292)	21.0 (1736)	18.3 (1072)	21.6 (2298)
	P	****	****	**	****	-
30m	Index	21.0 (221)	20.4 (139)	18.0 (181)	19.2 (418)	26.5 (40)
	Control	13.0 (820)	13.5 (841)	14.6 (1150)	12.1(680)	15.0 (1458)
	P	****	****	**	****	***
42m	Index	17.0 (164)	17.7 (129)	14.7 (158)	16.1 (371)	19.5 (29)
	Control	12.1 (792)	12.0 (779)	12.9 (14)	11.3 (670)	13.2 (1303)
	P	****	****	**	****	*

*P< 0.05; ** P < 0.01; *** P < 0.001; **** P < 0.0001

Supplementary Table 6. Comparison of the prevalence of **pus/sticky mucus leaking from ears more than once** among index children (i.e. with high levels of autistic traits or diagnosed autism) and control children (i.e. all who were not in the index category).

**Age**		**SCDC % (n)**	**Coherence % (n)**	**Sociability % (n)**	**Repetitive % (n)**	**Autism % (n)**
18m	Index	2.8 (27)	4.7 (34)	3.8 (40)	3.2 (73)	5.3 (8)
	Control	2.8 (184)	2.6 (169)	3.0 (250)	2.7 (159)	3.0 (322)
	P	-	***	-	-	-
30m	Index	3.9 (37)	5.1 (36)	4.4 (46)	4.0 (90)	10.3 (16)
	Control	3.2 (207)	3.0 (189)	3.3 (263)	3.1(177)	6.6 (349)
	P	-	**	-	-	***
42m	Index	8.3 (80)	10.3 (75)	7.2 (77)	7.3 (167)	15.4 (23)
	Control	5.2 (342)	5.2 (339)	5.7 (471)	5.3 (311)	5.8 (575)
	P	****	****	-	****	****
3-4 y	Index	4.8 (44)	4.7 (33)	5.6 (55)	4.0 (90)	9.6 (14)
	Control	3.2 (207)	3.2 (200)	3.2 (245)	3.1 (181)	3.5 (324)
	P	**	**	**	-	**

*P< 0.05; ** P < 0.01; *** P < 0.001; **** P < 0.0001

For peer review only

Supplementary Table 7. Comparison of the prevalence of **hearing reported to be worse during a cold** among index children (i.e. with high levels of autistic traits or diagnosed autism) and control children (i.e. all who were not in the index category).

Age		SCDC % (n)	Coherence % (n)	Sociability % (n)	Repetitive % (n)	Autism % (n)
18m	Index	20.2 (173)	21.9 (137)	17.7 (165)	18.5 (369)	29.9 (41)
	Control	14.4 (842)	14.6 (844)	15.4 (1143)	13.9 (736)	15.4 (1485)
	P	****	****	-	****	****
30m	Index	21.5 (148)	25.8 (133)	16.5 (127)	20.1 (319)	37.9 (44)
	Control	14.1 (682)	14.1 (673)	15.6 (946)	13.1(574)	15.2 (1136)
	P	****	****	-	****	****
42m	Index	37.1 (354)	41.4 (297)	31.6 (335)	36.3 (827)	52.4 (77)
	Control	29.9 (1943)	29.3 (1880)	30.5 (2512)	28.6 (1676)	30.5 (2989)
	P	****	****	-	****	****

*P< 0.05; ** P < 0.01; *** P < 0.001; **** P < 0.0001

Supplementary Table 8. Comparison of the prevalence of **rarely listening** among index children (i.e. with high levels of autistic traits or diagnosed autism) and control children (i.e. all who were not in the index category).

Age		SCDC % (n)	Coherence % (n)	Sociability % (n)	Repetitive % (n)	Autism % (n)
18m	Index	4.2 (42)	3.9 (29)	3.8 (40)	3.0 (70)	5.1 (8)
	Control	1.6 (108)	1.6 (103)	1.9 (162)	1.7 (98)	2.1 (235)
	P	****	****	*	*	*
30m	Index	4.6 (44)	6.4 (45)	5.1 (53)	4.1 (91)	14.4 (22)
	Control	1.6 (101)	1.5 (95)	1.7 (139)	1.4 (80)	2.1 (213)
	P	****	****	****	****	****
42m	Index	7.7 (75)	8.9 (65)	6.7 (72)	5.5 (126)	20.2 (30)
	Control	2.5 (165)	2.3 (148)	2.8 (235)	2.3 (135)	3.1 (306)
	P	****	****	****	****	****

*P< 0.05; ** P < 0.01; *** P < 0.001; **** P < 0.0001

For peer review only

STROBE Statement—Checklist of items that should be included in reports of ***cohort studies***

	Item No	Recommendation	Page No
**Title and abstract**	1	(*a*) Indicate the study's design with a commonly used term in the title or the abstract<br>(*b*) Provide in the abstract an informative and balanced summary of what was done and what was found	1<br>2
**Introduction**			
Background/rationale	2	Explain the scientific background and rationale for the investigation being reported	4
Objectives	3	State specific objectives, including any prespecified hypotheses	5
**Methods**			
Study design	4	Present key elements of study design early in the paper	5
Setting	5	Describe the setting, locations, and relevant dates, including periods of recruitment, exposure, follow-up, and data collection	5
Participants	6	(*a*) Give the eligibility criteria, and the sources and methods of selection of participants. Describe methods of follow-up<br>(*b*) For matched studies, give matching criteria and number of exposed and unexposed	6
Variables	7	Clearly define all outcomes, exposures, predictors, potential confounders, and effect modifiers. Give diagnostic criteria, if applicable	6-8
Data sources/ measurement	8*	For each variable of interest, give sources of data and details of methods of assessment (measurement). Describe comparability of assessment methods if there is more than one group	6-8
Bias	9	Describe any efforts to address potential sources of bias	8-9
Study size	10	Explain how the study size was arrived at	6
Quantitative variables	11	Explain how quantitative variables were handled in the analyses. If applicable, describe which groupings were chosen and why	6-9
Statistical methods	12	(*a*) Describe all statistical methods, including those used to control for confounding<br>(*b*) Describe any methods used to examine subgroups and interactions<br>(*c*) Explain how missing data were addressed<br>(*d*) If applicable, explain how loss to follow-up was addressed<br>(*e*) Describe any sensitivity analyses	9<br>N/A
**Results**			
Participants	13*	(a) Report numbers of individuals at each stage of study—eg numbers potentially eligible, examined for eligibility, confirmed eligible, included in the study, completing follow-up, and analysed<br>(b) Give reasons for non-participation at each stage<br>(c) Consider use of a flow diagram	6,13<br>N/A<br>N/A
Descriptive data	14*	(a) Give characteristics of study participants (eg demographic, clinical, social) and information on exposures and potential confounders<br>(b) Indicate number of participants with missing data for each variable of interest<br>(c) Summarise follow-up time (eg, average and total amount)	20<br>N/A
Outcome data	15*	Report numbers of outcome events or summary measures over time	6-8

For peer review only - http://bmjopen.bmj.com/site/about/guidelines.xhtml

Main results	16	(*a*) Give unadjusted estimates and, if applicable, confounder-adjusted estimates and their precision (eg, 95% confidence interval). Make clear which confounders were adjusted for and why they were included	9-11
		(*b*) Report category boundaries when continuous variables were categorized	20
		(*c*) If relevant, consider translating estimates of relative risk into absolute risk for a meaningful time period	
Other analyses	17	Report other analyses done—eg analyses of subgroups and interactions, and sensitivity analyses	N/A
**Discussion**			
Key results	18	Summarise key results with reference to study objectives	11-13
Limitations	19	Discuss limitations of the study, taking into account sources of potential bias or imprecision. Discuss both direction and magnitude of any potential bias	13
Interpretation	20	Give a cautious overall interpretation of results considering objectives, limitations, multiplicity of analyses, results from similar studies, and other relevant evidence	11-13
Generalisability	21	Discuss the generalisability (external validity) of the study results	14
**Other information**			
Funding	22	Give the source of funding and the role of the funders for the present study and, if applicable, for the original study on which the present article is based	14

*Give information separately for exposed and unexposed groups.

Note: An Explanation and Elaboration article discusses each checklist item and gives methodological background and published examples of transparent reporting. The STROBE checklist is best used in conjunction with this article (freely available on the Web sites of PLoS Medicine at http://www.plosmedicine.org/, Annals of Internal Medicine at http://www.annals.org/, and Epidemiology at http://www.epidem.com/). Information on the STROBE Initiative is available at http://www.strobe-statement.org.

For peer review only - http://bmjopen.bmj.com/site/about/guidelines.xhtml
